# Search for pair production of heavy particles decaying to a top quark and a gluon in the lepton+jets final state in proton–proton collisions at $\sqrt{s}=13\,\text {Te}\hspace{-.08em}\text {V}$

**DOI:** 10.1140/epjc/s10052-024-13729-y

**Published:** 2025-03-25

**Authors:** CMS Collaboration, A. Hayrapetyan, A. Tumasyan, W. Adam, J. W. Andrejkovic, L. Benato, T. Bergauer, S. Chatterjee, K. Damanakis, M. Dragicevic, P. S. Hussain, M. Jeitler, N. Krammer, A. Li, D. Liko, I. Mikulec, J. Schieck, R. Schöfbeck, D. Schwarz, M. Sonawane, W. Waltenberger, C.-E. Wulz, T. Janssen, T. Van Laer, P. Van Mechelen, N. Breugelmans, J. D’Hondt, S. Dansana, A. De Moor, M. Delcourt, F. Heyen, S. Lowette, I. Makarenko, D. Müller, S. Tavernier, M. Tytgat, G. P. Van Onsem, S. Van Putte, D. Vannerom, B. Bilin, B. Clerbaux, A. K. Das, I. De Bruyn, G. De Lentdecker, H. Evard, L. Favart, P. Gianneios, J. Jaramillo, A. Khalilzadeh, F. A. Khan, K. Lee, A. Malara, S. Paredes, M. A. Shahzad, L. Thomas, M. Vanden Bemden, C. Vander Velde, P. Vanlaer, M. De Coen, D. Dobur, G. Gokbulut, Y. Hong, J. Knolle, L. Lambrecht, D. Marckx, K. Mota Amarilo, K. Skovpen, N. Van Den Bossche, J. van der Linden, L. Wezenbeek, A. Benecke, A. Bethani, G. Bruno, C. Caputo, J. De Favereau De Jeneret, C. Delaere, I. S. Donertas, A. Giammanco, A. O. Guzel, Sa. Jain, V. Lemaitre, J. Lidrych, P. Mastrapasqua, T. T. Tran, S. Turkcapar, G. A. Alves, E. Coelho, G. Correia Silva, C. Hensel, T. Menezes De Oliveira, C. Mora Herrera, P. Rebello Teles, M. Soeiro, E. J. Tonelli Manganote, A. Vilela Pereira, W. L. Aldá Júnior, M. Barroso Ferreira Filho, H. Brandao Malbouisson, W. Carvalho, J. Chinellato, E. M. Da Costa, G. G. Da Silveira, D. De Jesus Damiao, S. Fonseca De Souza, R. Gomes De Souza, T. Laux Kuhn, M. Macedo, J. Martins, L. Mundim, H. Nogima, J. P. Pinheiro, A. Santoro, A. Sznajder, M. Thiel, C. A. Bernardes, L. Calligaris, T. R. Fernandez Perez Tomei, E. M. Gregores, I. Maietto Silverio, P. G. Mercadante, S. F. Novaes, B. Orzari, Sandra S. Padula, A. Aleksandrov, G. Antchev, R. Hadjiiska, P. Iaydjiev, M. Misheva, M. Shopova, G. Sultanov, A. Dimitrov, L. Litov, B. Pavlov, P. Petkov, A. Petrov, E. Shumka, S. Keshri, D. Laroze, S. Thakur, T. Cheng, T. Javaid, L. Yuan, Z. Hu, Z. Liang, J. Liu, G. M. Chen, H. S. Chen, M. Chen, F. Iemmi, C. H. Jiang, A. Kapoor, H. Liao, Z.-A. Liu, R. Sharma, J. N. Song, J. Tao, C. Wang, J. Wang, Z. Wang, H. Zhang, J. Zhao, A. Agapitos, Y. Ban, S. Deng, B. Guo, C. Jiang, A. Levin, C. Li, Q. Li, Y. Mao, S. Qian, S. J. Qian, X. Qin, X. Sun, D. Wang, H. Yang, L. Zhang, Y. Zhao, C. Zhou, S. Yang, Z. You, K. Jaffel, N. Lu, G. Bauer, B. Li, K. Yi, J. Zhang, Y. Li, Z. Lin, C. Lu, M. Xiao, C. Avila, D. A. Barbosa Trujillo, A. Cabrera, C. Florez, J. Fraga, J. A. Reyes Vega, F. Ramirez, C. Rendón, M. Rodriguez, A. A. Ruales Barbosa, J. D. Ruiz Alvarez, D. Giljanovic, N. Godinovic, D. Lelas, A. Sculac, M. Kovac, A. Petkovic, T. Sculac, P. Bargassa, V. Brigljevic, B. K. Chitroda, D. Ferencek, K. Jakovcic, A. Starodumov, T. Susa, A. Attikis, K. Christoforou, A. Hadjiagapiou, C. Leonidou, J. Mousa, C. Nicolaou, L. Paizanos, F. Ptochos, P. A. Razis, H. Rykaczewski, H. Saka, A. Stepennov, M. Finger, M. Finger Jr, A. Kveton, E. Ayala, E. Carrera Jarrin, H. Abdalla, Y. Assran, B. El-mahdy, M. Abdullah Al-Mashad, M. A. Mahmoud, K. Ehataht, M. Kadastik, T. Lange, S. Nandan, C. Nielsen, J. Pata, M. Raidal, L. Tani, C. Veelken, H. Kirschenmann, K. Osterberg, M. Voutilainen, S. Bharthuar, N. Bin Norjoharuddeen, E. Brücken, F. Garcia, P. Inkaew, K. T. S. Kallonen, T. Lampén, K. Lassila-Perini, S. Lehti, T. Lindén, M. Myllymäki, M.m. Rantanen, H. Siikonen, J. Tuominiemi, P. Luukka, H. Petrow, M. Besancon, F. Couderc, M. Dejardin, D. Denegri, J. L. Faure, F. Ferri, S. Ganjour, P. Gras, G. Hamel de Monchenault, M. Kumar, V. Lohezic, J. Malcles, F. Orlandi, L. Portales, A. Rosowsky, M. Ö. Sahin, A. Savoy-Navarro, P. Simkina, M. Titov, M. Tornago, F. Beaudette, G. Boldrini, P. Busson, A. Cappati, C. Charlot, M. Chiusi, T. D. Cuisset, F. Damas, O. Davignon, A. De Wit, I. T. Ehle, B. A. Fontana Santos Alves, S. Ghosh, A. Gilbert, R. Granier de Cassagnac, A. Hakimi, B. Harikrishnan, L. Kalipoliti, G. Liu, M. Nguyen, C. Ochando, R. Salerno, J. B. Sauvan, Y. Sirois, L. Urda Gómez, E. Vernazza, A. Zabi, A. Zghiche, J.-L. Agram, J. Andrea, D. Apparu, D. Bloch, J.-M. Brom, E. C. Chabert, C. Collard, S. Falke, U. Goerlach, R. Haeberle, A.-C. Le Bihan, M. Meena, O. Poncet, G. Saha, M. A. Sessini, P. Van Hove, P. Vaucelle, A. Di Florio, D. Amram, S. Beauceron, B. Blancon, G. Boudoul, N. Chanon, D. Contardo, P. Depasse, C. Dozen, H. El Mamouni, J. Fay, S. Gascon, M. Gouzevitch, C. Greenberg, G. Grenier, B. Ille, E. Jourd‘huy, I. B. Laktineh, M. Lethuillier, L. Mirabito, S. Perries, A. Purohit, M. Vander Donckt, P. Verdier, J. Xiao, A. Khvedelidze, I. Lomidze, Z. Tsamalaidze, V. Botta, S. Consuegra Rodríguez, L. Feld, K. Klein, M. Lipinski, D. Meuser, A. Pauls, D. Pérez Adán, N. Röwert, M. Teroerde, S. Diekmann, A. Dodonova, N. Eich, D. Eliseev, F. Engelke, J. Erdmann, M. Erdmann, P. Fackeldey, B. Fischer, T. Hebbeker, K. Hoepfner, F. Ivone, A. Jung, M.y. Lee, F. Mausolf, M. Merschmeyer, A. Meyer, S. Mukherjee, D. Noll, F. Nowotny, A. Pozdnyakov, Y. Rath, W. Redjeb, F. Rehm, H. Reithler, V. Sarkisovi, A. Schmidt, C. Seth, A. Sharma, J. L. Spah, A. Stein, F. Torres Da Silva De Araujo, S. Wiedenbeck, S. Zaleski, C. Dziwok, G. Flügge, T. Kress, A. Nowack, O. Pooth, A. Stahl, T. Ziemons, A. Zotz, H. Aarup Petersen, M. Aldaya Martin, J. Alimena, S. Amoroso, Y. An, J. Bach, S. Baxter, M. Bayatmakou, H. Becerril Gonzalez, O. Behnke, A. Belvedere, F. Blekman, K. Borras, A. Campbell, A. Cardini, C. Cheng, F. Colombina, G. Eckerlin, D. Eckstein, L. I. Estevez Banos, E. Gallo, A. Geiser, V. Guglielmi, M. Guthoff, A. Hinzmann, L. Jeppe, B. Kaech, M. Kasemann, C. Kleinwort, R. Kogler, M. Komm, D. Krücker, W. Lange, D. Leyva Pernia, K. Lipka, W. Lohmann, F. Lorkowski, R. Mankel, I.-A. Melzer-Pellmann, M. Mendizabal Morentin, A. B. Meyer, G. Milella, K. Moral Figueroa, A. Mussgiller, L. P. Nair, J. Niedziela, A. Nürnberg, Y. Otarid, J. Park, E. Ranken, A. Raspereza, D. Rastorguev, J. Rübenach, L. Rygaard, A. Saggio, M. Scham, S. Schnake, P. Schütze, C. Schwanenberger, D. Selivanova, K. Sharko, M. Shchedrolosiev, D. Stafford, F. Vazzoler, A. Ventura Barroso, R. Walsh, D. Wang, Q. Wang, K. Wichmann, L. Wiens, C. Wissing, Y. Yang, A. Zimermmane Castro Santos, A. Albrecht, S. Albrecht, M. Antonello, S. Bein, S. Bollweg, M. Bonanomi, P. Connor, K. El Morabit, Y. Fischer, E. Garutti, A. Grohsjean, J. Haller, D. Hundhausen, H. R. Jabusch, G. Kasieczka, P. Keicher, R. Klanner, W. Korcari, T. Kramer, C. c. Kuo, V. Kutzner, F. Labe, J. Lange, A. Lobanov, C. Matthies, L. Moureaux, M. Mrowietz, A. Nigamova, Y. Nissan, A. Paasch, K. J. Pena Rodriguez, T. Quadfasel, B. Raciti, M. Rieger, D. Savoiu, J. Schindler, P. Schleper, M. Schröder, J. Schwandt, M. Sommerhalder, H. Stadie, G. Steinbrück, A. Tews, B. Wiederspan, M. Wolf, S. Brommer, E. Butz, T. Chwalek, A. Dierlamm, A. Droll, U. Elicabuk, N. Faltermann, M. Giffels, A. Gottmann, F. Hartmann, R. Hofsaess, M. Horzela, U. Husemann, J. Kieseler, M. Klute, R. Koppenhöfer, O. Lavoryk, J. M. Lawhorn, M. Link, A. Lintuluoto, S. Maier, S. Mitra, M. Mormile, Th. Müller, M. Neukum, M. Oh, E. Pfeffer, M. Presilla, G. Quast, K. Rabbertz, B. Regnery, N. Shadskiy, I. Shvetsov, H. J. Simonis, L. Sowa, L. Stockmeier, K. Tauqeer, M. Toms, N. Trevisani, R. F. Von Cube, M. Wassmer, S. Wieland, F. Wittig, R. Wolf, X. Zuo, G. Anagnostou, G. Daskalakis, A. Kyriakis, A. Papadopoulos, A. Stakia, P. Kontaxakis, G. Melachroinos, Z. Painesis, I. Papavergou, I. Paraskevas, N. Saoulidou, K. Theofilatos, E. Tziaferi, K. Vellidis, I. Zisopoulos, G. Bakas, T. Chatzistavrou, G. Karapostoli, K. Kousouris, I. Papakrivopoulos, E. Siamarkou, G. Tsipolitis, A. Zacharopoulou, I. Bestintzanos, I. Evangelou, C. Foudas, C. Kamtsikis, P. Katsoulis, P. Kokkas, P. G. Kosmoglou Kioseoglou, N. Manthos, I. Papadopoulos, J. Strologas, C. Hajdu, D. Horvath, K. Márton, A. J. Rádl, F. Sikler, V. Veszpremi, M. Csanád, K. Farkas, A. Fehérkuti, M. M. A. Gadallah, Á. Kadlecsik, P. Major, G. Pásztor, G. I. Veres, L. Olah, B. Ujvari, G. Bencze, S. Czellar, J. Molnar, Z. Szillasi, T. Csorgo, F. Nemes, T. Novak, S. Bansal, S. B. Beri, V. Bhatnagar, G. Chaudhary, S. Chauhan, N. Dhingra, A. Kaur, A. Kaur, H. Kaur, M. Kaur, S. Kumar, T. Sheokand, J. B. Singh, A. Singla, A. Ahmed, A. Bhardwaj, A. Chhetri, B. C. Choudhary, A. Kumar, A. Kumar, M. Naimuddin, K. Ranjan, M. K. Saini, S. Saumya, S. Baradia, S. Barman, S. Bhattacharya, S. Das Gupta, S. Dutta, S. Dutta, S. Sarkar, M. M. Ameen, P. K. Behera, S. C. Behera, S. Chatterjee, G. Dash, P. Jana, P. Kalbhor, S. Kamble, J. R. Komaragiri, D. Kumar, T. Mishra, B. Parida, P. R. Pujahari, N. R. Saha, A. Sharma, A. K. Sikdar, R. K. Singh, P. Verma, S. Verma, A. Vijay, S. Dugad, G. B. Mohanty, M. Shelake, P. Suryadevara, A. Bala, S. Banerjee, R. M. Chatterjee, M. Guchait, Sh. Jain, A. Jaiswal, S. Kumar, G. Majumder, K. Mazumdar, S. Parolia, A. Thachayath, S. Bahinipati, C. Kar, D. Maity, P. Mal, K. Naskar, A. Nayak, S. Nayak, K. Pal, P. Sadangi, S. K. Swain, S. Varghese, D. Vats, S. Acharya, A. Alpana, S. Dube, B. Gomber, P. Hazarika, B. Kansal, A. Laha, B. Sahu, S. Sharma, K. Y. Vaish, H. Bakhshiansohi, A. Jafari, M. Zeinali, S. Bashiri, S. Chenarani, S. M. Etesami, Y. Hosseini, M. Khakzad, E. Khazaie, M. Mohammadi Najafabadi, S. Tizchang, M. Felcini, M. Grunewald, M. Abbrescia, A. Colaleo, D. Creanza, B. D’Anzi, N. De Filippis, M. De Palma, W. Elmetenawee, N. Ferrara, L. Fiore, G. Iaselli, L. Longo, M. Louka, G. Maggi, M. Maggi, I. Margjeka, V. Mastrapasqua, S. My, S. Nuzzo, A. Pellecchia, A. Pompili, G. Pugliese, R. Radogna, D. Ramos, A. Ranieri, L. Silvestris, F. M. Simone, Ü. Sözbilir, A. Stamerra, D. Troiano, R. Venditti, P. Verwilligen, A. Zaza, G. Abbiendi, C. Battilana, D. Bonacorsi, P. Capiluppi, A. Castro, F. R. Cavallo, M. Cuffiani, G. M. Dallavalle, T. Diotalevi, F. Fabbri, A. Fanfani, D. Fasanella, P. Giacomelli, L. Giommi, C. Grandi, L. Guiducci, S. Lo Meo, M. Lorusso, L. Lunerti, S. Marcellini, G. Masetti, F. L. Navarria, G. Paggi, A. Perrotta, F. Primavera, A. M. Rossi, S. Rossi Tisbeni, T. Rovelli, G. P. Siroli, S. Costa, A. Di Mattia, A. Lapertosa, R. Potenza, A. Tricomi, C. Tuve, P. Assiouras, G. Barbagli, G. Bardelli, B. Camaiani, A. Cassese, R. Ceccarelli, V. Ciulli, C. Civinini, R. D’Alessandro, E. Focardi, T. Kello, G. Latino, P. Lenzi, M. Lizzo, M. Meschini, S. Paoletti, A. Papanastassiou, G. Sguazzoni, L. Viliani, L. Benussi, S. Bianco, S. Meola, D. Piccolo, M. Alves Gallo Pereira, F. Ferro, E. Robutti, S. Tosi, A. Benaglia, F. Brivio, F. Cetorelli, F. De Guio, M. E. Dinardo, P. Dini, S. Gennai, R. Gerosa, A. Ghezzi, P. Govoni, L. Guzzi, M. T. Lucchini, M. Malberti, S. Malvezzi, A. Massironi, D. Menasce, L. Moroni, M. Paganoni, S. Palluotto, D. Pedrini, A. Perego, B. S. Pinolini, G. Pizzati, S. Ragazzi, T. Tabarelli de Fatis, S. Buontempo, A. Cagnotta, F. Carnevali, N. Cavallo, F. Fabozzi, A. O. M. Iorio, L. Lista, P. Paolucci, B. Rossi, R. Ardino, P. Azzi, N. Bacchetta, P. Bortignon, G. Bortolato, A. Bragagnolo, A. C. M. Bulla, R. Carlin, P. Checchia, T. Dorigo, F. Gasparini, U. Gasparini, S. Giorgetti, E. Lusiani, M. Margoni, G. Maron, A. T. Meneguzzo, M. Migliorini, J. Pazzini, P. Ronchese, R. Rossin, F. Simonetto, M. Tosi, A. Triossi, S. Ventura, M. Zanetti, P. Zotto, A. Zucchetta, G. Zumerle, A. Braghieri, S. Calzaferri, D. Fiorina, P. Montagna, V. Re, C. Riccardi, P. Salvini, I. Vai, P. Vitulo, S. Ajmal, M. E. Ascioti, G. M. Bilei, C. Carrivale, D. Ciangottini, L. Fanò, M. Magherini, V. Mariani, M. Menichelli, F. Moscatelli, A. Rossi, A. Santocchia, D. Spiga, T. Tedeschi, C. Aimè, C. A. Alexe, P. Asenov, P. Azzurri, G. Bagliesi, R. Bhattacharya, L. Bianchini, T. Boccali, E. Bossini, D. Bruschini, R. Castaldi, M. A. Ciocci, M. Cipriani, V. D’Amante, R. Dell’Orso, S. Donato, A. Giassi, F. Ligabue, A. C. Marini, D. Matos Figueiredo, A. Messineo, S. Mishra, V. K. Muraleedharan Nair Bindhu, M. Musich, F. Palla, A. Rizzi, G. Rolandi, S. Roy Chowdhury, T. Sarkar, A. Scribano, P. Spagnolo, R. Tenchini, G. Tonelli, N. Turini, F. Vaselli, A. Venturi, P. G. Verdini, C. Baldenegro Barrera, P. Barria, C. Basile, F. Cavallari, L. Cunqueiro Mendez, D. Del Re, E. Di Marco, M. Diemoz, F. Errico, R. Gargiulo, E. Longo, L. Martikainen, J. Mijuskovic, G. Organtini, F. Pandolfi, R. Paramatti, C. Quaranta, S. Rahatlou, C. Rovelli, F. Santanastasio, L. Soffi, V. Vladimirov, N. Amapane, R. Arcidiacono, S. Argiro, M. Arneodo, N. Bartosik, R. Bellan, A. Bellora, C. Biino, C. Borca, N. Cartiglia, M. Costa, R. Covarelli, N. Demaria, L. Finco, M. Grippo, B. Kiani, F. Legger, F. Luongo, C. Mariotti, L. Markovic, S. Maselli, A. Mecca, L. Menzio, P. Meridiani, E. Migliore, M. Monteno, R. Mulargia, M. M. Obertino, G. Ortona, L. Pacher, N. Pastrone, M. Pelliccioni, M. Ruspa, F. Siviero, V. Sola, A. Solano, A. Staiano, C. Tarricone, D. Trocino, G. Umoret, R. White, J. Babbar, S. Belforte, V. Candelise, M. Casarsa, F. Cossutti, K. De Leo, G. Della Ricca, S. Dogra, J. Hong, B. Kim, J. Kim, D. Lee, H. Lee, S. W. Lee, C. S. Moon, Y. D. Oh, M. S. Ryu, S. Sekmen, B. Tae, Y. C. Yang, M. S. Kim, G. Bak, P. Gwak, H. Kim, D. H. Moon, E. Asilar, J. Choi, D. Kim, T. J. Kim, J. A. Merlin, Y. Ryou, S. Choi, S. Han, B. Hong, K. Lee, K. S. Lee, S. Lee, J. Yoo, J. Goh, S. Yang, H. S. Kim, Y. Kim, S. Lee, J. Almond, J. H. Bhyun, J. Choi, J. Choi, W. Jun, J. Kim, Y. W. Kim, S. Ko, H. Kwon, H. Lee, J. Lee, J. Lee, B. H. Oh, S. B. Oh, H. Seo, U. K. Yang, I. Yoon, W. Jang, D. Y. Kang, Y. Kang, S. Kim, B. Ko, J. S. H. Lee, Y. Lee, I. C. Park, Y. Roh, I. J. Watson, S. Ha, K. Hwang, H. D. Yoo, M. Choi, M. R. Kim, H. Lee, Y. Lee, I. Yu, T. Beyrouthy, Y. Gharbia, F. Alazemi, K. Dreimanis, A. Gaile, C. Munoz Diaz, D. Osite, G. Pikurs, A. Potrebko, M. Seidel, D. Sidiropoulos Kontos, N. R. Strautnieks, M. Ambrozas, A. Juodagalvis, A. Rinkevicius, G. Tamulaitis, I. Yusuff, Z. Zolkapli, J. F. Benitez, A. Castaneda Hernandez, H. A. Encinas Acosta, L. G. Gallegos Maríñez, M. León Coello, J. A. Murillo Quijada, A. Sehrawat, L. Valencia Palomo, G. Ayala, H. Castilla-Valdez, H. Crotte Ledesma, E. De La Cruz-Burelo, I. Heredia-De La Cruz, R. Lopez-Fernandez, J. Mejia Guisao, C. A. Mondragon Herrera, A. Sánchez Hernández, C. Oropeza Barrera, D. L. Ramirez Guadarrama, M. Ramírez García, I. Bautista, I. Pedraza, H. A. Salazar Ibarguen, C. Uribe Estrada, I. Bubanja, N. Raicevic, P. H. Butler, A. Ahmad, M. I. Asghar, A. Awais, M. I. M. Awan, H. R. Hoorani, W. A. Khan, V. Avati, L. Grzanka, M. Malawski, H. Bialkowska, M. Bluj, M. Górski, M. Kazana, M. Szleper, P. Zalewski, K. Bunkowski, K. Doroba, A. Kalinowski, M. Konecki, J. Krolikowski, A. Muhammad, P. Fokow, K. Pozniak, W. Zabolotny, M. Araujo, D. Bastos, C. Beirão Da Cruz E Silva, A. Boletti, M. Bozzo, T. Camporesi, G. Da Molin, P. Faccioli, M. Gallinaro, J. Hollar, N. Leonardo, G. B. Marozzo, A. Petrilli, M. Pisano, J. Seixas, J. Varela, J. W. Wulff, P. Adzic, P. Milenovic, D. Devetak, M. Dordevic, J. Milosevic, L. Nadderd, V. Rekovic, J. Alcaraz Maestre, Cristina F. Bedoya, J. A. Brochero Cifuentes, Oliver M. Carretero, M. Cepeda, M. Cerrada, N. Colino, B. De La Cruz, A. Delgado Peris, A. Escalante Del Valle, D. Fernández Del Val, J. P. Fernández Ramos, J. Flix, M. C. Fouz, O. Gonzalez Lopez, S. Goy Lopez, J. M. Hernandez, M. I. Josa, J. Llorente Merino, C. Martin Perez, E. Martin Viscasillas, D. Moran, C. M. Morcillo Perez, Á. Navarro Tobar, C. Perez Dengra, A. Pérez-Calero Yzquierdo, J. Puerta Pelayo, I. Redondo, S. Sánchez Navas, J. Sastre, J. Vazquez Escobar, J. F. de Trocóniz, B. Alvarez Gonzalez, J. Cuevas, J. Fernandez Menendez, S. Folgueras, I. Gonzalez Caballero, P. Leguina, E. Palencia Cortezon, J. Prado Pico, C. Ramón Álvarez, V. Rodríguez Bouza, A. Soto Rodríguez, A. Trapote, C. Vico Villalba, P. Vischia, S. Bhowmik, S. Blanco Fernández, I. J. Cabrillo, A. Calderon, J. Duarte Campderros, M. Fernandez, G. Gomez, C. Lasaosa García, R. Lopez Ruiz, C. Martinez Rivero, P. Martinez Ruiz del Arbol, F. Matorras, P. Matorras Cuevas, E. Navarrete Ramos, J. Piedra Gomez, L. Scodellaro, I. Vila, J. M. Vizan Garcia, B. Kailasapathy, D. D. C. Wickramarathna, W. G. D. Dharmaratna, K. Liyanage, N. Perera, D. Abbaneo, C. Amendola, E. Auffray, G. Auzinger, J. Baechler, D. Barney, A. Bermúdez Martínez, M. Bianco, A. A. Bin Anuar, A. Bocci, L. Borgonovi, C. Botta, E. Brondolin, C. Caillol, G. Cerminara, N. Chernyavskaya, D. d’Enterria, A. Dabrowski, A. David, A. De Roeck, M. M. Defranchis, M. Deile, M. Dobson, G. Franzoni, W. Funk, S. Giani, D. Gigi, K. Gill, F. Glege, J. Hegeman, J. K. Heikkilä, B. Huber, V. Innocente, T. James, P. Janot, O. Kaluzinska, O. Karacheban, S. Laurila, P. Lecoq, E. Leutgeb, C. Lourenço, L. Malgeri, M. Mannelli, M. Matthewman, A. Mehta, F. Meijers, S. Mersi, E. Meschi, V. Milosevic, F. Monti, F. Moortgat, M. Mulders, I. Neutelings, S. Orfanelli, F. Pantaleo, G. Petrucciani, A. Pfeiffer, M. Pierini, H. Qu, D. Rabady, B. Ribeiro Lopes, F. Riti, M. Rovere, H. Sakulin, R. Salvatico, S. Sanchez Cruz, S. Scarfi, C. Schwick, M. Selvaggi, A. Sharma, K. Shchelina, P. Silva, P. Sphicas, A. G. Stahl Leiton, A. Steen, S. Summers, D. Treille, P. Tropea, D. Walter, J. Wanczyk, J. Wang, K. A. Wozniak, S. Wuchterl, P. Zehetner, P. Zejdl, W. D. Zeuner, T. Bevilacqua, L. Caminada, A. Ebrahimi, W. Erdmann, R. Horisberger, Q. Ingram, H. C. Kaestli, D. Kotlinski, C. Lange, M. Missiroli, L. Noehte, T. Rohe, A. Samalan, T. K. Aarrestad, M. Backhaus, G. Bonomelli, A. Calandri, C. Cazzaniga, K. Datta, P. De Bryas Dexmiers D‘archiac, A. De Cosa, G. Dissertori, M. Dittmar, M. Donegà, F. Eble, M. Galli, K. Gedia, F. Glessgen, C. Grab, N. Härringer, T. G. Harte, D. Hits, W. Lustermann, A.-M. Lyon, R. A. Manzoni, M. Marchegiani, L. Marchese, A. Mascellani, F. Nessi-Tedaldi, F. Pauss, V. Perovic, S. Pigazzini, B. Ristic, R. Seidita, J. Steggemann, A. Tarabini, D. Valsecchi, R. Wallny, C. Amsler, P. Bärtschi, M. F. Canelli, K. Cormier, M. Huwiler, W. Jin, A. Jofrehei, B. Kilminster, S. Leontsinis, S. P. Liechti, A. Macchiolo, P. Meiring, F. Meng, J. Motta, A. Reimers, P. Robmann, M. Senger, E. Shokr, F. Stäger, R. Tramontano, C. Adloff, D. Bhowmik, C. M. Kuo, W. Lin, P. K. Rout, P. C. Tiwari, L. Ceard, K. F. Chen, Z. g. Chen, A. De Iorio, W.-S. Hou, T. h. Hsu, Y. w. Kao, S. Karmakar, G. Kole, Y.y. Li, R.-S. Lu, E. Paganis, X.f. Su, J. Thomas-Wilsker, L. s. Tsai, D. Tsionou, H. y. Wu, E. Yazgan, C. Asawatangtrakuldee, N. Srimanobhas, V. Wachirapusitanand, Y. Maghrbi, D. Agyel, F. Boran, F. Dolek, I. Dumanoglu, E. Eskut, Y. Guler, E. Gurpinar Guler, C. Isik, O. Kara, A. Kayis Topaksu, U. Kiminsu, Y. Komurcu, G. Onengut, K. Ozdemir, A. Polatoz, B. Tali, U. G. Tok, E. Uslan, I. S. Zorbakir, G. Sokmen, M. Yalvac, B. Akgun, I. O. Atakisi, E. Gülmez, M. Kaya, O. Kaya, S. Tekten, A. Cakir, K. Cankocak, G. G. Dincer, S. Sen, O. Aydilek, B. Hacisahinoglu, I. Hos, B. Kaynak, S. Ozkorucuklu, O. Potok, H. Sert, C. Simsek, C. Zorbilmez, S. Cerci, B. Isildak, D. Sunar Cerci, T. Yetkin, A. Boyaryntsev, B. Grynyov, L. Levchuk, D. Anthony, J. J. Brooke, A. Bundock, F. Bury, E. Clement, D. Cussans, H. Flacher, M. Glowacki, J. Goldstein, H. F. Heath, M.-L. Holmberg, L. Kreczko, S. Paramesvaran, L. Robertshaw, V. J. Smith, K. Walkingshaw Pass, A. H. Ball, K. W. Bell, A. Belyaev, C. Brew, R. M. Brown, D. J. A. Cockerill, C. Cooke, A. Elliot, K. V. Ellis, K. Harder, S. Harper, J. Linacre, K. Manolopoulos, D. M. Newbold, E. Olaiya, D. Petyt, T. Reis, A. R. Sahasransu, G. Salvi, T. Schuh, C. H. Shepherd-Themistocleous, I. R. Tomalin, K. C. Whalen, T. Williams, I. Andreou, R. Bainbridge, P. Bloch, C. E. Brown, O. Buchmuller, C. A. Carrillo Montoya, G. S. Chahal, D. Colling, J. S. Dancu, I. Das, P. Dauncey, G. Davies, M. Della Negra, S. Fayer, G. Fedi, G. Hall, A. Howard, G. Iles, C. R. Knight, P. Krueper, J. Langford, K. H. Law, J. León Holgado, L. Lyons, A.-M. Magnan, B. Maier, S. Mallios, M. Mieskolainen, J. Nash, M. Pesaresi, P. B. Pradeep, B. C. Radburn-Smith, A. Richards, A. Rose, K. Savva, C. Seez, R. Shukla, A. Tapper, K. Uchida, G. P. Uttley, T. Virdee, M. Vojinovic, N. Wardle, D. Winterbottom, J. E. Cole, A. Khan, P. Kyberd, I. D. Reid, S. Abdullin, A. Brinkerhoff, E. Collins, M. R. Darwish, J. Dittmann, K. Hatakeyama, V. Hegde, J. Hiltbrand, B. McMaster, J. Samudio, S. Sawant, C. Sutantawibul, J. Wilson, R. Bartek, A. Dominguez, A. E. Simsek, S. S. Yu, B. Bam, A. Buchot Perraguin, R. Chudasama, S. I. Cooper, C. Crovella, S. V. Gleyzer, E. Pearson, C. U. Perez, P. Rumerio, E. Usai, R. Yi, A. Akpinar, C. Cosby, G. De Castro, Z. Demiragli, C. Erice, C. Fangmeier, C. Fernandez Madrazo, E. Fontanesi, D. Gastler, F. Golf, S. Jeon, J. O‘cain, I. Reed, J. Rohlf, K. Salyer, D. Sperka, D. Spitzbart, I. Suarez, A. Tsatsos, A. G. Zecchinelli, G. Barone, G. Benelli, D. Cutts, L. Gouskos, M. Hadley, U. Heintz, K. W. Ho, J. M. Hogan, T. Kwon, G. Landsberg, K. T. Lau, J. Luo, S. Mondal, T. Russell, S. Sagir, X. Shen, F. Simpson, M. Stamenkovic, N. Venkatasubramanian, S. Abbott, B. Barton, C. Brainerd, R. Breedon, H. Cai, M. Calderon De La Barca Sanchez, M. Chertok, M. Citron, J. Conway, P. T. Cox, R. Erbacher, F. Jensen, O. Kukral, G. Mocellin, M. Mulhearn, S. Ostrom, W. Wei, S. Yoo, F. Zhang, K. Adamidis, M. Bachtis, D. Campos, R. Cousins, A. Datta, G. Flores Avila, J. Hauser, M. Ignatenko, M. A. Iqbal, T. Lam, Y. f. Lo, E. Manca, A. Nunez Del Prado, D. Saltzberg, V. Valuev, R. Clare, J. W. Gary, G. Hanson, A. Aportela, A. Arora, J. G. Branson, S. Cittolin, S. Cooperstein, D. Diaz, J. Duarte, L. Giannini, Y. Gu, J. Guiang, R. Kansal, V. Krutelyov, R. Lee, J. Letts, M. Masciovecchio, F. Mokhtar, S. Mukherjee, M. Pieri, D. Primosch, M. Quinnan, B. V. Sathia Narayanan, V. Sharma, M. Tadel, E. Vourliotis, F. Würthwein, Y. Xiang, A. Yagil, A. Barzdukas, L. Brennan, C. Campagnari, K. Downham, C. Grieco, M. M. Hussain, J. Incandela, J. Kim, A. J. Li, P. Masterson, H. Mei, J. Richman, S. N. Santpur, U. Sarica, R. Schmitz, F. Setti, J. Sheplock, D. Stuart, T.Á. Vámi, S. Wang, X. Yan, D. Zhang, S. Bhattacharya, A. Bornheim, O. Cerri, A. Latorre, J. Mao, H. B. Newman, G. Reales Gutiérrez, M. Spiropulu, J. R. Vlimant, C. Wang, S. Xie, R. Y. Zhu, J. Alison, S. An, P. Bryant, M. Cremonesi, V. Dutta, T. Ferguson, T. A. Gómez Espinosa, A. Harilal, A. Kallil Tharayil, C. Liu, T. Mudholkar, S. Murthy, P. Palit, K. Park, M. Paulini, A. Roberts, A. Sanchez, W. Terrill, J. P. Cumalat, W. T. Ford, A. Hart, A. Hassani, G. Karathanasis, N. Manganelli, J. Pearkes, C. Savard, N. Schonbeck, K. Stenson, K. A. Ulmer, S. R. Wagner, N. Zipper, D. Zuolo, J. Alexander, S. Bright-Thonney, X. Chen, D. J. Cranshaw, J. Dickinson, J. Fan, X. Fan, S. Hogan, P. Kotamnives, J. Monroy, M. Oshiro, J. R. Patterson, M. Reid, A. Ryd, J. Thom, P. Wittich, R. Zou, M. Albrow, M. Alyari, O. Amram, G. Apollinari, A. Apresyan, L. A. T. Bauerdick, D. Berry, J. Berryhill, P. C. Bhat, K. Burkett, J. N. Butler, A. Canepa, G. B. Cerati, H. W. K. Cheung, F. Chlebana, G. Cummings, I. Dutta, V. D. Elvira, Y. Feng, J. Freeman, A. Gandrakota, Z. Gecse, L. Gray, D. Green, A. Grummer, S. Grünendahl, D. Guerrero, O. Gutsche, R. M. Harris, R. Heller, T. C. Herwig, J. Hirschauer, B. Jayatilaka, S. Jindariani, M. Johnson, U. Joshi, T. Klijnsma, B. Klima, K. H. M. Kwok, S. Lammel, C. Lee, D. Lincoln, R. Lipton, T. Liu, C. Madrid, K. Maeshima, C. Mantilla, D. Mason, P. McBride, P. Merkel, S. Mrenna, S. Nahn, J. Ngadiuba, D. Noonan, S. Norberg, V. Papadimitriou, N. Pastika, K. Pedro, C. Pena, F. Ravera, A. Reinsvold Hall, L. Ristori, M. Safdari, E. Sexton-Kennedy, N. Smith, A. Soha, L. Spiegel, S. Stoynev, J. Strait, L. Taylor, S. Tkaczyk, N. V. Tran, L. Uplegger, E. W. Vaandering, I. Zoi, C. Aruta, P. Avery, D. Bourilkov, P. Chang, V. Cherepanov, R. D. Field, C. Huh, E. Koenig, M. Kolosova, J. Konigsberg, A. Korytov, K. Matchev, N. Menendez, G. Mitselmakher, K. Mohrman, A. Muthirakalayil Madhu, N. Rawal, S. Rosenzweig, Y. Takahashi, J. Wang, T. Adams, A. Al Kadhim, A. Askew, S. Bower, R. Hashmi, R. S. Kim, S. Kim, T. Kolberg, G. Martinez, H. Prosper, P. R. Prova, M. Wulansatiti, R. Yohay, J. Zhang, B. Alsufyani, S. Butalla, S. Das, T. Elkafrawy, M. Hohlmann, E. Yanes, M. R. Adams, A. Baty, C. Bennett, R. Cavanaugh, R. Escobar Franco, O. Evdokimov, C. E. Gerber, M. Hawksworth, A. Hingrajiya, D. J. Hofman, J.h. Lee, D. S. Lemos, A. H. Merrit, C. Mills, S. Nanda, G. Oh, B. Ozek, D. Pilipovic, R. Pradhan, E. Prifti, T. Roy, S. Rudrabhatla, N. Singh, M. B. Tonjes, N. Varelas, M. A. Wadud, Z. Ye, J. Yoo, M. Alhusseini, D. Blend, K. Dilsiz, L. Emediato, G. Karaman, O. K. Köseyan, J.-P. Merlo, A. Mestvirishvili, O. Neogi, H. Ogul, Y. Onel, A. Penzo, C. Snyder, E. Tiras, B. Blumenfeld, L. Corcodilos, J. Davis, A. V. Gritsan, L. Kang, S. Kyriacou, P. Maksimovic, M. Roguljic, J. Roskes, S. Sekhar, M. Swartz, A. Abreu, L. F. Alcerro Alcerro, J. Anguiano, S. Arteaga Escatel, P. Baringer, A. Bean, Z. Flowers, D. Grove, J. King, G. Krintiras, M. Lazarovits, C. Le Mahieu, J. Marquez, M. Murray, M. Nickel, M. Pitt, S. Popescu, C. Rogan, C. Royon, S. Sanders, C. Smith, G. Wilson, B. Allmond, R. Gujju Gurunadha, A. Ivanov, K. Kaadze, Y. Maravin, J. Natoli, D. Roy, G. Sorrentino, A. Baden, A. Belloni, J. Bistany-riebman, Y. M. Chen, S. C. Eno, N. J. Hadley, S. Jabeen, R. G. Kellogg, T. Koeth, B. Kronheim, Y. Lai, S. Lascio, A. C. Mignerey, S. Nabili, C. Palmer, C. Papageorgakis, M. M. Paranjpe, E. Popova, A. Shevelev, L. Wang, J. Bendavid, I. A. Cali, P.c. Chou, M. D’Alfonso, J. Eysermans, C. Freer, G. Gomez-Ceballos, M. Goncharov, G. Grosso, P. Harris, D. Hoang, D. Kovalskyi, J. Krupa, L. Lavezzo, Y.-J. Lee, K. Long, C. Mcginn, A. Novak, M. I. Park, C. Paus, C. Reissel, C. Roland, G. Roland, S. Rothman, G. S. F. Stephans, Z. Wang, B. Wyslouch, T. J. Yang, B. Crossman, B. M. Joshi, C. Kapsiak, M. Krohn, D. Mahon, J. Mans, B. Marzocchi, M. Revering, R. Rusack, R. Saradhy, N. Strobbe, K. Bloom, D. R. Claes, G. Haza, J. Hossain, C. Joo, I. Kravchenko, A. Rohilla, J. E. Siado, W. Tabb, A. Vagnerini, A. Wightman, F. Yan, D. Yu, H. Bandyopadhyay, L. Hay, H. w. Hsia, I. Iashvili, A. Kalogeropoulos, A. Kharchilava, M. Morris, D. Nguyen, S. Rappoccio, H. Rejeb Sfar, A. Williams, P. Young, G. Alverson, E. Barberis, J. Bonilla, B. Bylsma, M. Campana, J. Dervan, Y. Haddad, Y. Han, I. Israr, A. Krishna, J. Li, M. Lu, G. Madigan, R. Mccarthy, D. M. Morse, V. Nguyen, T. Orimoto, A. Parker, L. Skinnari, E. Tsai, D. Wood, J. Bueghly, S. Dittmer, K. A. Hahn, D. Li, Y. Liu, M. Mcginnis, Y. Miao, D. G. Monk, M. H. Schmitt, A. Taliercio, M. Velasco, G. Agarwal, R. Band, R. Bucci, S. Castells, A. Das, R. Goldouzian, M. Hildreth, K. Hurtado Anampa, T. Ivanov, C. Jessop, K. Lannon, J. Lawrence, N. Loukas, L. Lutton, J. Mariano, N. Marinelli, I. Mcalister, T. McCauley, C. Mcgrady, C. Moore, Y. Musienko, H. Nelson, M. Osherson, A. Piccinelli, R. Ruchti, A. Townsend, Y. Wan, M. Wayne, H. Yockey, M. Zarucki, L. Zygala, A. Basnet, M. Carrigan, L. S. Durkin, C. Hill, M. Joyce, M. Nunez Ornelas, K. Wei, D. A. Wenzl, B. L. Winer, B. R. Yates, H. Bouchamaoui, K. Coldham, P. Das, G. Dezoort, P. Elmer, A. Frankenthal, B. Greenberg, N. Haubrich, K. Kennedy, G. Kopp, S. Kwan, D. Lange, A. Loeliger, D. Marlow, I. Ojalvo, J. Olsen, D. Stickland, C. Tully, L. H. Vage, S. Malik, R. Sharma, A. S. Bakshi, S. Chandra, R. Chawla, A. Gu, L. Gutay, M. Jones, A. W. Jung, A. M. Koshy, M. Liu, G. Negro, N. Neumeister, G. Paspalaki, S. Piperov, V. Scheurer, J. F. Schulte, M. Stojanovic, J. Thieman, A. K. Virdi, F. Wang, A. Wildridge, W. Xie, Y. Yao, J. Dolen, N. Parashar, A. Pathak, D. Acosta, T. Carnahan, K. M. Ecklund, P. J. Fernández Manteca, S. Freed, P. Gardner, F. J. M. Geurts, I. Krommydas, W. Li, J. Lin, O. Miguel Colin, B. P. Padley, R. Redjimi, J. Rotter, E. Yigitbasi, Y. Zhang, A. Bodek, P. de Barbaro, R. Demina, J. L. Dulemba, A. Garcia-Bellido, O. Hindrichs, A. Khukhunaishvili, N. Parmar, P. Parygin, R. Taus, B. Chiarito, J. P. Chou, S. V. Clark, D. Gadkari, Y. Gershtein, E. Halkiadakis, M. Heindl, C. Houghton, D. Jaroslawski, S. Konstantinou, I. Laflotte, A. Lath, R. Montalvo, K. Nash, J. Reichert, H. Routray, P. Saha, S. Salur, S. Schnetzer, S. Somalwar, R. Stone, S. A. Thayil, S. Thomas, J. Vora, H. Wang, D. Ally, A. G. Delannoy, S. Fiorendi, S. Higginbotham, T. Holmes, A. R. Kanuganti, N. Karunarathna, L. Lee, E. Nibigira, S. Spanier, D. Aebi, M. Ahmad, T. Akhter, K. Androsov, O. Bouhali, R. Eusebi, J. Gilmore, T. Huang, T. Kamon, H. Kim, S. Luo, R. Mueller, D. Overton, D. Rathjens, A. Safonov, N. Akchurin, J. Damgov, N. Gogate, A. Hussain, Y. Kazhykarim, K. Lamichhane, S. W. Lee, A. Mankel, T. Peltola, I. Volobouev, E. Appelt, Y. Chen, S. Greene, A. Gurrola, W. Johns, R. Kunnawalkam Elayavalli, A. Melo, F. Romeo, P. Sheldon, S. Tuo, J. Velkovska, J. Viinikainen, B. Cardwell, H. Chung, B. Cox, J. Hakala, R. Hirosky, A. Ledovskoy, C. Neu, S. Bhattacharya, P. E. Karchin, A. Aravind, S. Banerjee, K. Black, T. Bose, E. Chavez, S. Dasu, P. Everaerts, C. Galloni, H. He, M. Herndon, A. Herve, C. K. Koraka, A. Lanaro, R. Loveless, J. Madhusudanan Sreekala, A. Mallampalli, A. Mohammadi, S. Mondal, G. Parida, L. Pétré, D. Pinna, A. Savin, V. Shang, V. Sharma, W. H. Smith, D. Teague, H. F. Tsoi, W. Vetens, A. Warden, S. Afanasiev, V. Alexakhin, D. Budkouski, I. Golutvin, I. Gorbunov, V. Karjavine, V. Korenkov, A. Lanev, A. Malakhov, V. Matveev, V. Palichik, V. Perelygin, M. Savina, V. Shalaev, S. Shmatov, S. Shulha, V. Smirnov, O. Teryaev, N. Voytishin, B. S. Yuldashev, A. Zarubin, I. Zhizhin, G. Gavrilov, V. Golovtcov, Y. Ivanov, V. Kim, P. Levchenko, V. Murzin, V. Oreshkin, D. Sosnov, V. Sulimov, L. Uvarov, A. Vorobyev, Yu. Andreev, A. Dermenev, S. Gninenko, N. Golubev, A. Karneyeu, D. Kirpichnikov, M. Kirsanov, N. Krasnikov, I. Tlisova, A. Toropin, T. Aushev, K. Ivanov, V. Gavrilov, N. Lychkovskaya, A. Nikitenko, V. Popov, A. Zhokin, R. Chistov, M. Danilov, S. Polikarpov, V. Andreev, M. Azarkin, M. Kirakosyan, A. Terkulov, E. Boos, V. Bunichev, M. Dubinin, L. Dudko, V. Klyukhin, O. Kodolova, S. Obraztsov, M. Perfilov, S. Petrushanko, V. Savrin, P. Volkov, G. Vorotnikov, V. Blinov, T. Dimova, A. Kozyrev, O. Radchenko, Y. Skovpen, V. Kachanov, D. Konstantinov, S. Slabospitskii, A. Uzunian, A. Babaev, V. Borshch, D. Druzhkin

**Affiliations:** 1 Yerevan Physics Institute, Yerevan, Armenia; 2 Institut für Hochenergiephysik, Vienna, Austria; 3 Universiteit Antwerpen, Antwerpen, Belgium; 4 Vrije Universiteit Brussel, Brussel, Belgium; 5 Université Libre de Bruxelles, Bruxelles, Belgium; 6 Ghent University, Ghent, Belgium; 7 Université Catholique de Louvain, Louvain-la-Neuve, Belgium; 8 Centro Brasileiro de Pesquisas Fisicas, Rio de Janeiro, Brazil; 9 Universidade do Estado do Rio de Janeiro, Rio de Janeiro, Brazil; 10 Universidade Estadual Paulista, Universidade Federal do ABC, São Paulo, Brazil; 11 Institute for Nuclear Research and Nuclear Energy, Bulgarian Academy of Sciences, Sofia, Bulgaria; 12 University of Sofia, Sofia, Bulgaria; 13 Instituto De Alta Investigación, Universidad de Tarapacá, Casilla 7 D, Arica, Chile; 14 Beihang University, Beijing, China; 15 Department of Physics, Tsinghua University, Beijing, China; 16 Institute of High Energy Physics, Beijing, China; 17 State Key Laboratory of Nuclear Physics and Technology, Peking University, Beijing, China; 18 Guangdong Provincial Key Laboratory of Nuclear Science and Guangdong-Hong Kong Joint Laboratory of Quantum Matter, South China Normal University, Guangzhou, China; 19 Sun Yat-Sen University, Guangzhou, China; 20 University of Science and Technology of China, Hefei, China; 21 Nanjing Normal University, Nanjing, China; 22 Institute of Modern Physics and Key Laboratory of Nuclear Physics and Ion-beam Application (MOE)-Fudan University, Shanghai, China; 23 Zhejiang University, Hangzhou, Zhejiang, China; 24 Universidad de Los Andes, Bogota, Colombia; 25 Universidad de Antioquia, Medellin, Colombia; 26 University of Split, Faculty of Electrical Engineering, Mechanical Engineering and Naval Architecture, Split, Croatia; 27 University of Split, Faculty of Science, Split, Croatia; 28 Institute Rudjer Boskovic, Zagreb, Croatia; 29 University of Cyprus, Nicosia, Cyprus; 30 Charles University, Prague, Czech Republic; 31 Escuela Politecnica Nacional, Quito, Ecuador; 32 Universidad San Francisco de Quito, Quito, Ecuador; 33 Academy of Scientific Research and Technology of the Arab Republic of Egypt, Egyptian Network of High Energy Physics, Cairo, Egypt; 34 Center for High Energy Physics (CHEP-FU), Fayoum University, El-Fayoum, Egypt; 35 National Institute of Chemical Physics and Biophysics, Tallinn, Estonia; 36 Department of Physics, University of Helsinki, Helsinki, Finland; 37 Helsinki Institute of Physics, Helsinki, Finland; 38 Lappeenranta-Lahti University of Technology, Lappeenranta, Finland; 39 IRFU, CEA, Université Paris-Saclay, Gif-sur-Yvette, France; 40 Laboratoire Leprince-Ringuet, CNRS/IN2P3, Ecole Polytechnique, Institut Polytechnique de Paris, Palaiseau, France; 41 Université de Strasbourg, CNRS, IPHC UMR 7178, Strasbourg, France; 42 Centre de Calcul de l’Institut National de Physique Nucleaire et de Physique des Particules, CNRS/IN2P3, Villeurbanne, France; 43 Institut de Physique des 2 Infinis de Lyon (IP2I ), Villeurbanne, France; 44 Georgian Technical University, Tbilisi, Georgia; 45 I. Physikalisches Institut, RWTH Aachen University, Aachen, Germany; 46 III. Physikalisches Institut A, RWTH Aachen University, Aachen, Germany; 47 III. Physikalisches Institut B, RWTH Aachen University, Aachen, Germany; 48 Deutsches Elektronen-Synchrotron, Hamburg, Germany; 49 University of Hamburg, Hamburg, Germany; 50 Karlsruher Institut fuer Technologie, Karlsruhe, Germany; 51 Institute of Nuclear and Particle Physics (INPP), NCSR Demokritos, Aghia Paraskevi, Greece; 52 National and Kapodistrian University of Athens, Athens, Greece; 53 National Technical University of Athens, Athens, Greece; 54 University of Ioánnina, Ioannina, Greece; 55 HUN-REN Wigner Research Centre for Physics, Budapest, Hungary; 56 MTA-ELTE Lendület CMS Particle and Nuclear Physics Group, Eötvös Loránd University, Budapest, Hungary; 57 Faculty of Informatics, University of Debrecen, Debrecen, Hungary; 58 HUN-REN ATOMKI-Institute of Nuclear Research, Debrecen, Hungary; 59 Karoly Robert Campus, MATE Institute of Technology, Gyongyos, Hungary; 60 Panjab University, Chandigarh, India; 61 University of Delhi, Delhi, India; 62 Saha Institute of Nuclear Physics, HBNI, Kolkata, India; 63 Indian Institute of Technology Madras, Madras, India; 64 Tata Institute of Fundamental Research-A, Mumbai, India; 65 Tata Institute of Fundamental Research-B, Mumbai, India; 66 National Institute of Science Education and Research, An OCC of Homi Bhabha National Institute, Bhubaneswar, Odisha India; 67 Indian Institute of Science Education and Research (IISER), Pune, India; 68 Isfahan University of Technology, Isfahan, Iran; 69 Institute for Research in Fundamental Sciences (IPM), Tehran, Iran; 70 University College Dublin, Dublin, Ireland; 71 INFN Sezione di Bari, Università di Bari, Politecnico di Bari, Bari, Italy; 72 INFN Sezione di Bologna, Università di Bologna, Bologna, Italy; 73 INFN Sezione di Catania, Università di Catania, Catania, Italy; 74 INFN Sezione di Firenze, Università di Firenze, Firenze, Italy; 75 INFN Laboratori Nazionali di Frascati, Frascati, Italy; 76 INFN Sezione di Genova, Università di Genova, Genoa, Italy; 77 INFN Sezione di Milano-Bicocca, Università di Milano-Bicocca, Milan, Italy; 78 INFN Sezione di Napoli, Università di Napoli ’Federico II’, Napoli, Italy; Università della Basilicata, Potenza, Italy; Scuola Superiore Meridionale (SSM), Naples, Italy; 79 INFN Sezione di Padova, Università di Padova, Padova, Italy; Università di Trento, Trento, Italy; 80 INFN Sezione di Pavia, Università di Pavia, Pavia, Italy; 81 INFN Sezione di Perugia, Università di Perugia, Perugia, Italy; 82 INFN Sezione di Pisa, Università di Pisa, Scuola Normale Superiore di Pisa, Pisa, Italy; Università di Siena, Siena, Italy; 83 INFN Sezione di Roma, Sapienza Università di Roma, Rome, Italy; 84 INFN Sezione di Torino, Università di Torino, Torino, Italy; Università del Piemonte Orientale, Novara, Italy; 85 INFN Sezione di Trieste, Università di Trieste, Trieste, Italy; 86 Kyungpook National University, Daegu, Korea; 87 Department of Mathematics and Physics - GWNU, Gangneung, Korea; 88 Chonnam National University, Institute for Universe and Elementary Particles, Kwangju, Korea; 89 Hanyang University, Seoul, Korea; 90 Korea University, Seoul, Korea; 91 Kyung Hee University, Department of Physics, Seoul, Korea; 92 Sejong University, Seoul, Korea; 93 Seoul National University, Seoul, Korea; 94 University of Seoul, Seoul, Korea; 95 Yonsei University, Department of Physics, Seoul, Korea; 96 Sungkyunkwan University, Suwon, Korea; 97 College of Engineering and Technology, American University of the Middle East (AUM), Dasman, Kuwait; 98 Department of Physics, Kuwait University-College of Science, Safat, Kuwait; 99 Riga Technical University, Riga, Latvia; 100 University of Latvia (LU), Riga, Latvia; 101 Vilnius University, Vilnius, Lithuania; 102 National Centre for Particle Physics, Universiti Malaya, Kuala Lumpur, Malaysia; 103 Universidad de Sonora (UNISON), Hermosillo, Mexico; 104 Centro de Investigacion y de Estudios Avanzados del IPN, Mexico City, Mexico; 105 Universidad Iberoamericana, Mexico City, Mexico; 106 Benemerita Universidad Autonoma de Puebla, Puebla, Mexico; 107 University of Montenegro, Podgorica, Montenegro; 108 University of Canterbury, Christchurch, New Zealand; 109 National Centre for Physics, Quaid-I-Azam University, Islamabad, Pakistan; 110 AGH University of Krakow, Kraków, Poland; 111 National Centre for Nuclear Research, Swierk, Poland; 112 Institute of Experimental Physics, Faculty of Physics, University of Warsaw, Warsaw, Poland; 113 Warsaw University of Technology, Warsaw, Poland; 114 Laboratório de Instrumentação e Física Experimental de Partículas, Lisbon, Portugal; 115 Faculty of Physics, University of Belgrade, Belgrade, Serbia; 116 VINCA Institute of Nuclear Sciences, University of Belgrade, Belgrade, Serbia; 117 Centro de Investigaciones Energéticas Medioambientales y Tecnológicas (CIEMAT), Madrid, Spain; 118 Universidad Autónoma de Madrid, Madrid, Spain; 119 Instituto Universitario de Ciencias y Tecnologías Espaciales de Asturias (ICTEA), Universidad de Oviedo, Oviedo, Spain; 120 Instituto de Física de Cantabria (IFCA), CSIC-Universidad de Cantabria, Santander, Spain; 121 University of Colombo, Colombo, Sri Lanka; 122 University of Ruhuna, Department of Physics, Matara, Sri Lanka; 123 CERN, European Organization for Nuclear Research, Geneva, Switzerland; 124 Paul Scherrer Institut, Villigen, Switzerland; 125 ETH Zurich-Institute for Particle Physics and Astrophysics (IPA), Zurich, Switzerland; 126 Universität Zürich, Zurich, Switzerland; 127 National Central University, Chung-Li, Taiwan; 128 National Taiwan University (NTU), Taipei, Taiwan; 129 High Energy Physics Research Unit, Department of Physics, Faculty of Science, Chulalongkorn University, Bangkok, Thailand; 130 Tunis El Manar University, Tunis, Tunisia; 131 Physics Department Science and Art Faculty, Çukurova University, Adana, Turkey; 132 Physics Department, Middle East Technical University, Ankara, Turkey; 133 Bogazici University, Istanbul, Turkey; 134 Istanbul Technical University, Istanbul, Turkey; 135 Istanbul University, Istanbul, Turkey; 136 Yildiz Technical University, Istanbul, Turkey; 137 Institute for Scintillation Materials of National Academy of Science of Ukraine, Kharkiv, Ukraine; 138 National Science Centre, Kharkiv Institute of Physics and Technology, Kharkiv, Ukraine; 139 University of Bristol, Bristol, UK; 140 Rutherford Appleton Laboratory, Didcot, UK; 141 Imperial College, London, UK; 142 Brunel University, Uxbridge, UK; 143 Baylor University, Waco, TX USA; 144 Catholic University of America, Washington, DC USA; 145 The University of Alabama, Tuscaloosa, AL USA; 146 Boston University, Boston, MA USA; 147 Brown University, Providence, RI USA; 148 University of California, Davis, Davis, CA USA; 149 University of California, Los Angeles, CA USA; 150 University of California, Riverside, Riverside, CA USA; 151 University of California, San Diego, La Jolla, CA USA; 152 Department of Physics, University of California, Santa Barbara, Santa Barbara, CA USA; 153 California Institute of Technology, Pasadena, CA USA; 154 Carnegie Mellon University, Pittsburgh, PA USA; 155 University of Colorado Boulder, Boulder, CO USA; 156 Cornell University, Ithaca, NY USA; 157 Fermi National Accelerator Laboratory, Batavia, IL USA; 158 University of Florida, Gainesville, FL USA; 159 Florida State University, Tallahassee, FL USA; 160 Florida Institute of Technology, Melbourne, FL USA; 161 University of Illinois Chicago, Chicago, IL USA; 162 The University of Iowa, Iowa City, IA USA; 163 Johns Hopkins University, Baltimore, MD USA; 164 The University of Kansas, Lawrence, KS USA; 165 Kansas State University, Manhattan, KS USA; 166 University of Maryland, College Park, MD USA; 167 Massachusetts Institute of Technology, Cambridge, MA USA; 168 University of Minnesota, Minneapolis, MN USA; 169 University of Nebraska-Lincoln, Lincoln, NE USA; 170 State University of New York at Buffalo, Buffalo, NY USA; 171 Northeastern University, Boston, MA USA; 172 Northwestern University, Evanston, IL USA; 173 University of Notre Dame, Notre Dame, IN USA; 174 The Ohio State University, Columbus, OH USA; 175 Princeton University, Princeton, NJ USA; 176 University of Puerto Rico, Mayaguez, PR USA; 177 Purdue University, West Lafayette, IN USA; 178 Purdue University Northwest, Hammond, IN USA; 179 Rice University, Houston, TX USA; 180 University of Rochester, Rochester, NY USA; 181 Rutgers, The State University of New Jersey, Piscataway, NJ USA; 182 University of Tennessee, Knoxville, TN USA; 183 Texas A&M University, College Station, TX USA; 184 Texas Tech University, Lubbock, TX USA; 185 Vanderbilt University, Nashville, TN USA; 186 University of Virginia, Charlottesville, VA USA; 187 Wayne State University, Detroit, MI USA; 188 University of Wisconsin-Madison, Madison, WI USA; 189 Authors affiliated with an institute or an international laboratory covered by a cooperation agreement with CERN, Geneva, Switzerland; 190 Yerevan State University, Yerevan, Armenia; 191 TU Wien, Vienna, Austria; 192 Ghent University, Ghent, Belgium; 193 Universidade do Estado do Rio de Janeiro, Rio de Janeiro, Brazil; 194 FACAMP - Faculdades de Campinas, Sao Paulo, Brazil; 195 Universidade Estadual de Campinas, Campinas, Brazil; 196 Federal University of Rio Grande do Sul, Porto Alegre, Brazil; 197 University of Chinese Academy of Sciences, Beijing, China; 198 China Center of Advanced Science and Technology, Beijing, China; 199 University of Chinese Academy of Sciences, Beijing, China; 200 China Spallation Neutron Source, Guangdong, China; 201 Henan Normal University, Xinxiang, China; 202 University of Shanghai for Science and Technology, Shanghai, China; 203 The University of Iowa, Iowa City, USA; 204 an institute or an international laboratory covered by a cooperation agreement with CERN, Geneva, Switzerland; 205 Cairo University, Cairo, Egypt; 206 Suez University, Suez, Egypt; 207 British University in Egypt, Cairo, Egypt; 208 Purdue University, West Lafayette, IN USA; 209 Université de Haute Alsace, Mulhouse, France; 210 Istinye University, Istanbul, Turkey; 211 The University of the State of Amazonas, Manaus, Brazil; 212 University of Hamburg, Hamburg, Germany; 213 RWTH Aachen University, III. Physikalisches Institut A, Aachen, Germany; 214 Bergische University Wuppertal (BUW), Wuppertal, Germany; 215 Brandenburg University of Technology, Cottbus, Germany; 216 Forschungszentrum Jülich, Juelich, Germany; 217 CERN, European Organization for Nuclear Research, Geneva, Switzerland; 218 HUN-REN ATOMKI-Institute of Nuclear Research, Debrecen, Hungary; 219 Universitatea Babes-Bolyai-Facultatea de Fizica, Cluj-Napoca, Romania; 220 MTA-ELTE Lendület CMS Particle and Nuclear Physics Group, Eötvös Loránd University, Budapest, Hungary; 221 HUN-REN Wigner Research Centre for Physics, Budapest, Hungary; 222 Physics Department, Faculty of Science, Assiut University, Assiut, Egypt; 223 Punjab Agricultural University, Ludhiana, India; 224 University of Visva-Bharati, Santiniketan, India; 225 Indian Institute of Science (IISc), Bangalore, India; 226 Amity University Uttar Pradesh, Noida, India; 227 IIT Bhubaneswar, Bhubaneswar, India; 228 Institute of Physics, Bhubaneswar, India; 229 University of Hyderabad, Hyderabad, India; 230 Deutsches Elektronen-Synchrotron, Hamburg, Germany; 231 Isfahan University of Technology, Isfahan, Iran; 232 Sharif University of Technology, Tehran, Iran; 233 Department of Physics, University of Science and Technology of Mazandaran, Behshahr, Iran; 234 Department of Physics, Faculty of Science, Arak University, Arak, Iran; 235 Helwan University, Cairo, Egypt; 236 Italian National Agency for New Technologies, Energy and Sustainable Economic Development, Bologna, Italy; 237 Centro Siciliano di Fisica Nucleare e di Struttura Della Materia, Catania, Italy; 238 Università degli Studi Guglielmo Marconi, Rome, Italy; 239 Scuola Superiore Meridionale, Università di Napoli ’Federico II’, Naples, Italy; 240 Fermi National Accelerator Laboratory, Batavia, IL USA; 241 Laboratori Nazionali di Legnaro dell’INFN, Legnaro, Italy; 242 Consiglio Nazionale delle Ricerche - Istituto Officina dei Materiali, Perugia, Italy; 243 Department of Applied Physics, Faculty of Science and Technology, Universiti Kebangsaan Malaysia, Bangi, Malaysia; 244 Consejo Nacional de Ciencia y Tecnología, Mexico City, Mexico; 245 Trincomalee Campus, Eastern University, Nilaveli, Sri Lanka; 246 Saegis Campus, Nugegoda, Sri Lanka; 247 National and Kapodistrian University of Athens, Athens, Greece; 248 Ecole Polytechnique Fédérale Lausanne, Lausanne, Switzerland; 249 University of Vienna, Vienna, Austria; 250 Universität Zürich, Zurich, Switzerland; 251 Stefan Meyer Institute for Subatomic Physics, Vienna, Austria; 252 Laboratoire d’Annecy-le-Vieux de Physique des Particules, IN2P3-CNRS, Annecy-le-Vieux, France; 253 Near East University, Research Center of Experimental Health Science, Mersin, Turkey; 254 Konya Technical University, Konya, Turkey; 255 Izmir Bakircay University, Izmir, Turkey; 256 Adiyaman University, Adiyaman, Turkey; 257 Bozok Universitetesi Rektörlügü, Yozgat, Turkey; 258 Marmara University, Istanbul, Turkey; 259 Milli Savunma University, Istanbul, Turkey; 260 Kafkas University, Kars, Turkey; 261 Istanbul Okan University, Istanbul, Turkey; 262 Hacettepe University, Ankara, Turkey; 263 Erzincan Binali Yildirim University, Erzincan, Turkey; 264 Istanbul University - Cerrahpasa, Faculty of Engineering, Istanbul, Turkey; 265 Yildiz Technical University, Istanbul, Turkey; 266 School of Physics and Astronomy, University of Southampton, Southampton, UK; 267 IPPP Durham University, Durham, UK; 268 Monash University, Faculty of Science, Clayton, Australia; 269 Università di Torino, Turin, Italy; 270 Bethel University, St. Paul, MN USA; 271 Karamanoğlu Mehmetbey University, Karaman, Turkey; 272 California Institute of Technology, Pasadena, CA USA; 273 United States Naval Academy, Annapolis, MD USA; 274 Ain Shams University, Cairo, Egypt; 275 Bingol University, Bingol, Turkey; 276 Georgian Technical University, Tbilisi, Georgia; 277 Sinop University, Sinop, Turkey; 278 Erciyes University, Kayseri, Turkey; 279 Horia Hulubei National Institute of Physics and Nuclear Engineering (IFIN-HH), Bucharest, Romania; 280 another institute or international laboratory covered by a cooperation agreement with CERN, Geneva, Switzerland; 281 Texas A&M University at Qatar, Doha, Qatar; 282 Kyungpook National University, Daegu, Korea; 283 another institute or international laboratory covered by a cooperation agreement with CERN, Geneva, Switzerland; 284 Institute of Nuclear Physics of the Uzbekistan Academy of Sciences, Tashkent, Uzbekistan; 285 Northeastern University, Boston, MA USA; 286 Imperial College, London, UK; 287 Yerevan Physics Institute, Yerevan, Armenia; 288 Universiteit Antwerpen, Antwerpen, Belgium; 289 CERN, Geneva, Switzerland

## Abstract

A search is presented for the pair production of new heavy resonances, each decaying into a top quark (t) or antiquark and a gluon (g). The analysis uses data recorded with the CMS detector from proton–proton collisions at a center-of-mass energy of 13$\,\text {Te}\hspace{-.08em}\text {V}$ at the LHC, corresponding to an integrated luminosity of 138$\,\text {fb}^{-1}$. Events with one muon or electron, multiple jets, and missing transverse momentum are selected. After using a deep neural network to enrich the data sample with signal-like events, distributions in the scalar sum of the transverse momenta of all reconstructed objects are analyzed in the search for a signal. No significant deviations from the standard model prediction are found. Upper limits at 95% confidence level are set on the product of cross section and branching fraction squared for the pair production of excited top quarks in the $\text {t}^{*} \rightarrow {\text {t}} {\text {g}}$ decay channel. The upper limits range from 120 to 0.8$\,\text {fb}$ for a $\text {t}^{*}$with spin-1/2 and from 15 to 1.0$\,\text {fb}$ for a $\text {t}^{*}$with spin-3/2. These correspond to mass exclusion limits up to 1050 and 1700$\,\text {Ge}\hspace{-.08em}\text {V}$ for spin-1/2 and spin-3/2 $\text {t}^{*}$particles, respectively. These are the most stringent limits to date on the existence of $\text {t}^{*} \rightarrow {\text {t}} {\text {g}}$ resonances.

## Introduction

The standard model (SM) of particle physics has proven to be a highly accurate and successful theory. It describes the properties of all elementary particles and their interactions with a small number of free parameters. However, several observations indicate that it is not a complete theory and has to be extended. For example, the naturalness problem arises because of quadratically divergent contributions to the Higgs boson mass from the top quark and the W and Z bosons. In the SM, an unnaturally large fine-tuning is required to explain the measured Higgs boson mass of around 125$\,\text {Ge}\hspace{-.08em}\text {V}$ [1–3]. This motivates searches for possible extensions of the SM, particularly for theoretical models proposing that the top quark is not a fundamental but a composite particle [4–10]. A composite top quark would result in excited states that could be observed at the CERN LHC, providing a way to confirm or exclude these models. Excited top quarks $\text {t}^{*}$are predicted to decay instantly after their production into a top quark by the radiation of excess energy in the form of a gluon or a photon, $\text {t}^{*} \rightarrow {\text {t}} {\text {g}}$ or $\text {t}^{*} \rightarrow {\text {t}} {\upgamma }$. Other models of physics beyond the SM propose the existence of vector-like fermionic top quark partners, for example little Higgs models [11–14], models with extra dimensions [15–17], or composite Higgs models [18]. Usually, searches for these types of models are performed targeting decays including W, Z, or Higgs bosons [19–25]. However, in the case of a small mixing between the top quark partner and the top quark, these decay modes are suppressed and the quantum loop induced decays $\text {t}^{*} \rightarrow {\text {t}} {\text {g}}$ and $\text {t}^{*} \rightarrow {\text {t}} {\upgamma }$ become dominant [26]. Top quark partners can provide an elegant solution to the naturalness problem, introducing additional terms cancelling out the quadratic divergences [27].

In this article, we present a search for the pair production of heavy excited top quarks or top quark partners, generically labeled $\text {t}^{*}$in the following. We target the $\text {t}^{*} \bar{{\text {t}}}^{*} \rightarrow {\text {t}} {\text {g}} {{\text {t}}} {\text {g}}$ channel with an experimental signature that is independent of which underlying theory is predicting it. The $\text {t}^{*}$is predicted to carry the same weak isospin, color and weak hypercharge as the top quark, but could exist with different spins: spin-1/2 or spin-3/2 [26]. For a spin-1/2 $\text {t}^{*}$, the field can be described in a similar way to those of heavy SM quarks. The spin-3/2 case is described by a Rarita–Schwinger Lagrangian [28], adapting the Dirac equation to a spin-3/2 particle. Assuming equal couplings to the strong and electroweak sectors compared with the SM top quark leads to a branching fraction $\mathcal {B}$ of 97% for the decay $\text {t}^{*} \rightarrow {\text {t}} {\text {g}}$ in both spin scenarios [26]. This motivates a search where both $\text {t}^{*}$decay into a top quark and a gluon.

**Fig. 1:**
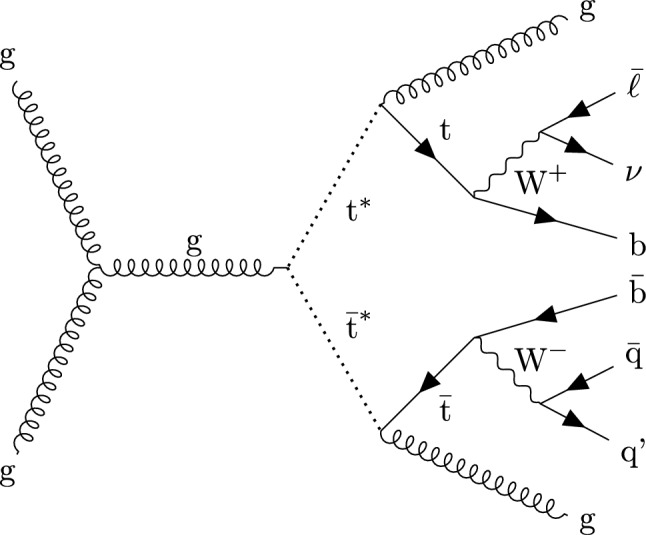
Representative Feynman diagram of the signal process at leading order

We search for the $\text {t}^{*} \bar{{\text {t}}}^{*} \rightarrow {\text {t}} {\text {g}} {{\text {t}}} {\text {g}}$ process in final states containing a single lepton, a neutrino, four light jets from two gluons and two light quarks, and two jets originating from b quarks. A representative Feynman diagram of a signal process is shown in Fig. 1. Data collected between 2016 and 2018 with the CMS detector in proton–proton (${\text {p}} {\text {p}}$) collisions at a center-of-mass energy of 13$\,\text {Te}\hspace{-.08em}\text {V}$ at the LHC are used in this analysis, corresponding to an integrated luminosity of 138$\,\text {fb}^{-1}$. Previous searches in the same final state have been performed by the CMS Collaboration using 8$\,\text {Te}\hspace{-.08em}\text {V}$ [29] and 13$\,\text {Te}\hspace{-.08em}\text {V}$ [30] ${\text {p}} {\text {p}}$ data. In comparison to the previous 13$\,\text {Te}\hspace{-.08em}\text {V}$ result, which is based on data corresponding to an integrated luminosity of 35.9$\,\text {fb}^{-1}$, the analysis presented in this article achieves a substantial increase in sensitivity from the larger data set and from improved analysis techniques. In particular, machine-learning techniques are used to suppress backgrounds from known SM processes, and a more inclusive observable for the signal extraction results in an improved signal efficiency compared to the previous 13$\,\text {Te}\hspace{-.08em}\text {V}$ analysis [30].

Tabulated results are provided in the HEPData record for this analysis [31].

## The CMS detector and simulated samples

The central feature of the CMS apparatus is a superconducting solenoid of 6$\,\text {m}$ internal diameter, providing a magnetic field of 3.8$\,\text {T}$. Within the solenoid volume are a silicon pixel and strip tracker, a lead tungstate crystal electromagnetic calorimeter (ECAL), and a brass and scintillator hadron calorimeter (HCAL), each composed of a barrel and two endcap sections. Forward calorimeters extend the pseudorapidity ($\eta$) coverage provided by the barrel and endcap detectors. Muons are measured in gas-ionization detectors embedded in the steel flux-return yoke outside the solenoid. More detailed descriptions of the CMS detector, together with a definition of the coordinate system used and the relevant kinematic variables, can be found in Refs. [32, 33].

Events of interest are selected using a two-tiered trigger system. The first level, composed of custom hardware processors, uses information from the calorimeters and muon detectors to select events at a rate of around 100$\,\text {kHz}$ within a fixed latency of 4$\,\mu \text {s}$ [34]. The second level, known as the high-level trigger (HLT), consists of a farm of processors running a version of the full event reconstruction software optimized for fast processing, and reduces the event rate to around 1$\,\text {kHz}$ before data storage [35].

Considering both the spin-1/2 and spin-3/2 scenarios, we produce Monte Carlo (MC) simulated samples of the ${\text {p}} {\text {p}} \rightarrow \text {t}^{*} \bar{{\text {t}}}^{*}$ signal process at leading order (LO) in perturbative quantum chromodynamics (QCD), using MadGraph 5_amc@nlo  2.6.5 [36]. We consider different values of resonance masses $m_{\text {t}^{*}}$ between 700 and 3000$\,\text {Ge}\hspace{-.08em}\text {V}$, in steps of 100$\,\text {Ge}\hspace{-.08em}\text {V}$ below 2000$\,\text {Ge}\hspace{-.08em}\text {V}$ and in steps of 250$\,\text {Ge}\hspace{-.08em}\text {V}$ above. The interactions with the SM particles are simulated using an effective field theory (EFT) [26, 37], with the assumption that the $\text {t}^{*}$couplings in the strong and electroweak sectors are identical to the top quark couplings. The predicted production cross sections for the pair production of spin-3/2 $\text {t}^{*}$particles is larger than for spin-1/2 particles of the same mass, as the cross section for spin-3/2 grows with energy as $\hat{s}^3$ [37], where $\hat{s}$ is the partonic center-of-mass energy squared. This violates unitarity at high energies, but is regulated by some large scale of new physics $\Lambda \gg m_{\text {t}^{*}}$ at which the EFT approach becomes invalid. Differences in the kinematic distributions of $\text {t}^{*}$systems with different spins are observed in angular correlations, as well as in the $\text {t}^{*}$momenta and the invariant mass of the $\text {t}^{*} \bar{{\text {t}}}^{*}$ system, where the momenta and mass are larger on average for spin-3/2 compared to spin-1/2 fermions. These spin-dependent differences become smaller with increasing $m_{\text {t}^{*}}$.

Relevant SM background processes for this search are top quark pair (t t) and single top quark production, as well as the production of a W boson in association with jets (W +jets). Less important background processes include the production of two weak gauge bosons (diboson), the production of multiple jets from the strong interaction (QCD multijet) and the Drell–Yan process. The t t production is simulated at next-to-LO (NLO) with powheg  v2 [38–42]. The cross section of the t t process is corrected to a prediction at next-to-NLO (NNLO) precision, using a next-to-next-to-leading-logarithmic soft-gluon approximation, obtained with the Top++ 2.0 program [43]. The production of single top quarks is simulated at NLO using powheg for the ${\text {t}} {W}$ and *t* channels, and MadGraph 5_amc@nlo for the *s* channel. The W +jets production is simulated at LO with four additional partons with MadGraph 5_amc@nlo. Diboson, QCD multijet production, and the Drell–Yan process are simulated at LO with pythia  8.240 [44]. For all generated samples, the NNPDF 3.1 [45] NNLO parton distribution function (PDF) sets are used.

For all generated collision events, parton shower and hadronization are simulated using pythia with the CP5 tune [46]. The CMS detector response is simulated using Geant4 [47]. Additional inelastic ${\text {p}} {\text {p}}$ collision events are simulated using pythia and superimposed on simulated events to model the effect of additional ${\text {p}} {\text {p}}$ collisions within the same or adjacent bunch crossings (pileup). We use a total inelastic cross section of 69.2$\,\text {mb}$ [48] to estimate the expected number of ${\text {p}} {\text {p}}$ interactions per bunch crossing and correct the simulation to match the corresponding distribution to that observed in data.

## Event reconstruction

A particle-flow (PF) algorithm [49] aims to reconstruct and identify each particle in an event, with an optimized combination of information from the various elements of the CMS detector.

The energy of muons is obtained from the curvature of the corresponding track. These are measured with a reconstruction and identification efficiency greater than 96%. For muons with transverse momentum $p_{\text {T}}$ up to 100$\,\text {Ge}\hspace{-.08em}\text {V}$, a relative transverse momentum resolution of 1% in the barrel and 3% in the endcaps is obtained. The $p_{\text {T}}$ resolution in the barrel is better than 7% for muons with $p_{\text {T}}$ up to 1$\,\text {Te}\hspace{-.08em}\text {V}$ [50]. A set of selection criteria, different for low-$p_{\text {T}}$ [51] and high-$p_{\text {T}}$ [52] muons, is used to select prompt muons. Scale factors are used to correct for observed differences between data and simulation in the muon reconstruction and selection efficiencies.

The energy of electrons is determined from a combination of the electron momentum at the primary interaction vertex as determined by the tracker, the energy of the corresponding ECAL cluster, and the energy sum of all bremsstrahlung photons spatially compatible with originating from the electron track [53]. The momentum resolution for electrons with $p_{\text {T}} \approx 45\,\text {Ge}\hspace{-.08em}\text {V}$ from ${Z} \rightarrow {{e}}{{e}}$ decays ranges from 1.6 to 5%. It is generally better in the barrel region than in the endcaps, and also depends on the bremsstrahlung energy emitted by the electron as it traverses the material in front of the ECAL [53, 54]. A multivariate selection with a 90% efficiency to identify prompt electrons is used. Correction scale factors ensure that the electron reconstruction and selection efficiency is well modeled in MC simulation.

For both muons and electrons, we define an isolation variable $I_{\text {rel}}$ as the $p_{\text {T}}$ sum of nearby PF candidates relative to the lepton $p_{\text {T}}$, after accounting for pileup contributions [50, 53]. For the calculation of $I_{\text {rel}}$, PF candidates within $\Delta {R} < 0.4$ and 0.3 for muons and electrons, respectively, are considered. The angular distance $\Delta {R}$ between two PF candidates *i* and *j* is defined as $\Delta {R} = \sqrt{\smash [b]{ (\Delta \eta _{i,j})^2 + (\Delta \phi _{i,j})^2 }}$, where $\Delta \eta _{i,j}$ and $\Delta \phi _{i,j}$ denote the distances in pseudorapidity and azimuth, respectively.

The energy of photons is obtained from the ECAL measurement [53]. The energy of charged hadrons is determined from a combination of their momentum measured in the tracker and the matching ECAL and HCAL energy deposits, corrected for the response function of the calorimeters to hadronic showers. The energy of neutral hadrons is obtained from the corresponding corrected ECAL and HCAL energies. Two different types of hadron jets are reconstructed from PF candidates, referred to as small-radius and variable-radius jets.

Small-radius jets are clustered using the anti-$k_{\text {T}}$ algorithm [55] as implemented in the FastJet package [56] with an angular distance parameter of 0.4. Jet momentum is determined as the vectorial sum of all particle momenta in the jet, and is found from simulation to be, on average, within 5 to 10% of the true momentum over the entire $p_{\text {T}}$ spectrum and detector acceptance. Pileup interactions can contribute additional tracks and calorimetric energy depositions, increasing the apparent jet momentum. The pileup-per-particle identification (PUPPI) algorithm [57, 58] is used to mitigate the effect of pileup at the reconstructed-particle level, using local shape information, event pileup properties, and tracking information. Jet energy corrections are derived from simulation studies so that the average measured energy of jets becomes identical to the truth information from the simulation. In situ measurements of the momentum balance in dijet, ${\upgamma }$+jet, Z +jet, and multijet events are used to determine any residual differences between the jet energy scale in data and in simulation, and appropriate corrections are made [59]. Additional selection criteria are applied to each jet to remove jets potentially dominated by instrumental effects or reconstruction failures [58].

The second jet reconstruction algorithm, used to reconstruct variable-radius jets, is the Heavy Object Tagger with Variable *R* (HOTVR) algorithm [60], which is also implemented in FastJet. It combines jet clustering with subjet finding and a soft-cluster rejection. Resulting jets have an angular size between 0.1 and 1.5 in the jet distance parameter, where the active jet area [61] decreases with increasing momentum. This jet reconstruction algorithm has been developed for the reconstruction of fully hadronic decays of Lorentz-boosted top quarks, where each decay results in a single jet reconstructed with an optimal jet size [62]. The top quark momentum, and therefore its Lorentz boost, varies depending on $m_{\text {t}^{*}}$ in this search. Variable-radius jets are thus better suited to capture the kinematic properties of the top quarks with reduced dependence on the assumed signal mass [63]. Jets originating from gluons of the $\text {t}^{*}$decay are reconstructed with the HOTVR algorithm as well, where the variable size of these jets helps to capture wide-angle radiation, which would result in out-of-cone effects for small-radius jets. The substructure of variable-radius jets is analyzed using the *N*-subjettiness variables $\tau _N$, which provide a measure of the degree to which jets contain *N* or fewer localized regions of high energy density [64, 65]. Pileup is accounted for using the PUPPI algorithm, following a similar procedure to that used for the small-radius jets. Momentum corrections are applied to the individual subjets of each HOTVR jet. Recombination of the corrected subjets gives the corrected variable-radius jet. This procedure was first applied in Ref. [66] and has been validated again for this analysis.

Jets originating from the fragmentation of b quarks are identified using the multiclassification algorithm DeepJet [67–69], based on a deep neural network (DNN). It uses information from jet constituents, charged and neutral particles, secondary vertices, and global event variables to define a score for each jet. This score quantifies how likely the jet is to have originated from the decay of a b quark. Small-radius with a DeepJet score above a certain threshold, corresponding to a 1% misidentification probability of light-quark and gluon jets, are referred to as b jets. This working point has an efficiency between 70 and 80% for correctly identifying b jets, depending on the data-taking era. Differences between data and simulation in the shape of the DeepJet score distribution are accounted for by applying correction factors, and a secondary correction ensures that no change in normalization is introduced.

The missing transverse momentum vector ${\textbf{p}}_{\text {T}}^{\hspace{1.66656pt}\text {miss}}$ is computed as the negative vector sum of the transverse momenta of all PF candidates in an event, and its magnitude is denoted as $p^{\textrm{miss}}_{\textrm{T}}$ [70]. The PUPPI algorithm is applied to reduce the pileup dependence of the ${\textbf{p}}_{\text {T}}^{\hspace{1.66656pt}\text {miss}}$ observable. The ${\textbf{p}}_{\text {T}}^{\hspace{1.66656pt}\text {miss}}$ is modified to account for corrections to the energy scale of the jets in the event.

## Event selection

We use HLT algorithms requiring the presence of a single muon, electron, or photon. These select events with at least one muon having $p_{\text {T}}$ above 50$\,\text {Ge}\hspace{-.08em}\text {V}$ or an electron with $p_{\text {T}}$ above 115$\,\text {Ge}\hspace{-.08em}\text {V}$, with no isolation requirements. Complementary triggers requiring an isolated lepton extend the reach of the analysis to lower $p_{\text {T}}$, requiring a muon with $p_{\text {T}}$ of at least 24 or 27$\,\text {Ge}\hspace{-.08em}\text {V}$ in 2016 or 2017–2018, or an electron with $p_{\text {T}}$ of at least 27, 35, or 32$\,\text {Ge}\hspace{-.08em}\text {V}$ in the years 2016, 2017, and 2018, respectively. The different $p_{\text {T}}$ thresholds were introduced because of different instantaneous luminosities during the three years of data taking. To recover inefficiencies at high electron $p_{\text {T}}$, events selected using photon triggers with a photon with $p_{\text {T}}$ larger than 175 or 200$\,\text {Ge}\hspace{-.08em}\text {V}$ in 2016 or 2017–2018, respectively, are considered as well. The muon trigger efficiency exceeds 90% over the full momentum range, and the electron trigger efficiency is greater than 80% and increases with momentum. The same trigger selection criteria are applied to simulated events, and scale factors ensure that the trigger efficiency in simulation matches that in data.

A further correction factor is applied to simulated events to account for a trigger inefficiency in 2016 and 2017, caused by a timing shift in the ECAL. An issue in some HCAL modules during 2018 resulted in a loss of efficiency in a specific $\eta$-$\phi$ region of the detector. Therefore, any event in data that has a muon, electron, or jet in the affected region during this period in time is rejected. The effect is accounted for in MC simulations using event weights.

To select events matching the expected topology of the signal process, we require events to have a single lepton $\ell$ (muon or electron) within $|\eta | < 2.4$, and $p_{\text {T}} > 30$ or 40$\,\text {Ge}\hspace{-.08em}\text {V}$ for muons or electrons, respectively. Events are assigned to a muon or an electron channel based on the flavor of the selected lepton. Muons or electrons with $p_{\text {T}}$ smaller than 55 or 120$\,\text {Ge}\hspace{-.08em}\text {V}$, respectively, are referred to as low-$p_{\text {T}}$ leptons. Low-$p_{\text {T}}$ muons need to fulfill $I_{\text {rel}} < 0.15$. For low-$p_{\text {T}}$ electrons, isolation is ensured by including relevant variables in the multivariate electron selection. High-$p_{\text {T}}$ leptons with $p_{\text {T}}$ above these thresholds are required to satisfy one of two looser isolation criteria: either $\Delta {R} (\ell , \text {jet}) > 0.4$ or $p_{\text {T,rel}} > 25\,\text {Ge}\hspace{-.08em}\text {V}$, to suppress contributions from QCD multijet production [71]. The angular distance $\Delta {R} (\ell , \text {jet})$ is calculated between the selected lepton and the closest small-radius jet in the $\eta$-$\phi$ plane, and $p_{\text {T,rel}}$ is the $p_{\text {T}}$ component of the lepton perpendicular to the axis of the closest small-radius jet.

For a hadronically decaying top quark, we require at least one variable-radius jet with $p_{\text {T}} > 200\,\text {Ge}\hspace{-.08em}\text {V}$ and $|\eta | < 2.4$, aiming to reconstruct the decay products in a single jet. Additionally, events must contain at least four small-radius jets with $p_{\text {T}} > 30\,\text {Ge}\hspace{-.08em}\text {V}$ and $|\eta | < 2.4$, at least one of which must be a b jet. In this selection, the variable-radius jets and small-radius jets are treated separately and are independent of each other. We do not test for a potential overlap between the small-radius and variable-radius jets in an event. Furthermore, $p^{\textrm{miss}}_{\textrm{T}} > 50\,\text {Ge}\hspace{-.08em}\text {V}$ is required to account for the neutrino from the W boson decay.

A scalar momentum sum variable $S_{\text {T}}$ is defined as

1 $$\begin{aligned} S_{\text {T}} = {p_{\text {T}}}^{\ell } + p^{\textrm{miss}}_{\textrm{T}} + \sum _{ \begin{array}{c} i \end{array} } {p_{\text {T},i}}^{\text {jet}}, \end{aligned}$$

where the sum runs over all variable-radius jets with $p_{\text {T}} > 200\,\text {Ge}\hspace{-.08em}\text {V}$ and $|\eta | < 2.4$. This variable is useful for discriminating signal from SM background events, as $\text {t}^{*} \bar{{\text {t}}}^{*}$ production results in a higher value of $S_{\text {T}}$ on average. In Sect. 8, we use the measured distribution in $S_{\text {T}}$ for the statistical interpretation of the results. All events are required to have $S_{\text {T}} > 500\,\text {Ge}\hspace{-.08em}\text {V}$ to further reduce trigger inefficiency effects from events with low-$p_{\text {T}}$ leptons and jets.

**Fig. 2:**
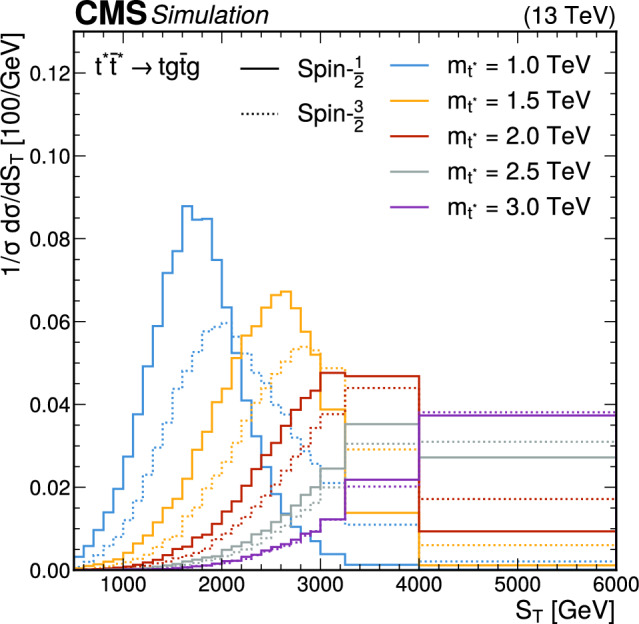
Distributions in $S_{\text {T}}$ for $\text {t}^{*} \bar{{\text {t}}}^{*}$ signal samples with different simulated values of $m_{\text {t}^{*}}$, for spin-1/2 (solid lines) and spin-3/2 (dashed lines) resonances. Each signal distribution is normalized to 100

Depending on the spin and mass of the $\text {t}^{*}$, around 50% of signal events remain after the above requirements, with higher efficiency for spin-3/2 compared to spin-1/2 $\text {t}^{*}$, and increasing efficiency towards higher $m_{\text {t}^{*}}$. In contrast, the contribution of SM background processes is substantially reduced, with efficiencies below 1%. In Fig. 2 we show the $S_{\text {T}}$ distributions of various simulated signal samples. The spin-3/2 signals feature a peak in $S_{\text {T}}$ around $2m_{\text {t}^{*}}$, shifted to smaller values for spin-1/2 signals. The spin-3/2 signals have higher $S_{\text {T}}$ on average, a difference that is more pronounced at lower $\text {t}^{*}$masses. This behavior results from higher $\text {t}^{*}$momentum and invariant mass of the $\text {t}^{*} \bar{{\text {t}}}^{*}$ system for spin-3/2 signals compared to spin-1/2. Because the SM background contributions monotonically decrease with increasing $S_{\text {T}}$, we expect better sensitivity for spin-3/2 signals compared to spin-1/2. These differences become smaller for higher $\text {t}^{*}$masses.

## Event classification

We use a DNN to classify events as signal like and background like. It is designed to output a single DNN score $s_{\text {DNN}}$, where signal-like events are given a high score and background-like events a low score. We pass 33 input features to the DNN, which are derived from the reconstructed PF candidates in signal events:

- $p_{\text {T}}$, $\eta$, $\phi$, and $I_{\text {rel}}$ of the lepton,
- $p_{\text {T}}$, $\eta$, $\phi$, the number of subjets, $\tau _1$, $\tau _2$, and $\tau _3$ of the three $p_{\text {T}}$-leading variable-radius jets,
- $p_{\text {T}}$, $\eta$, $\phi$ and the DeepJet score of the small-radius jet with the highest DeepJet score,
- $p^{\textrm{miss}}_{\textrm{T}}$ and $\phi$ of ${\textbf{p}}_{\text {T}}^{\hspace{1.66656pt}\text {miss}}$, and
- the multiplicities of the small-radius and variable-radius jets in the event.

Zero padding is used in case features cannot be defined, for example for events containing less than three variable-radius jets. We find that jet multiplicity and substructure variables are especially relevant for the DNN performance, together with information on the variable-radius jet that is third in the $p_{\text {T}}$ hierarchy. The DNN uses a fully connected feed-forward architecture with four hidden layers and 25 nodes each. We trained it on simulated t t events and a balanced mixture of spin-1/2 signal events with $m_{\text {t}^{*}}$ between 700 and 3000$\,\text {Ge}\hspace{-.08em}\text {V}$. We verified that no $m_{\text {t}^{*}}$ bias arises, by investigating the effect of repeating the training with one $m_{\text {t}^{*}}$ sample removed. The DNN is used for both spin scenarios, as we observe no substantial sensitivity increase when testing a separate, dedicated DNN for the spin-3/2 signals.

**Fig. 3:**
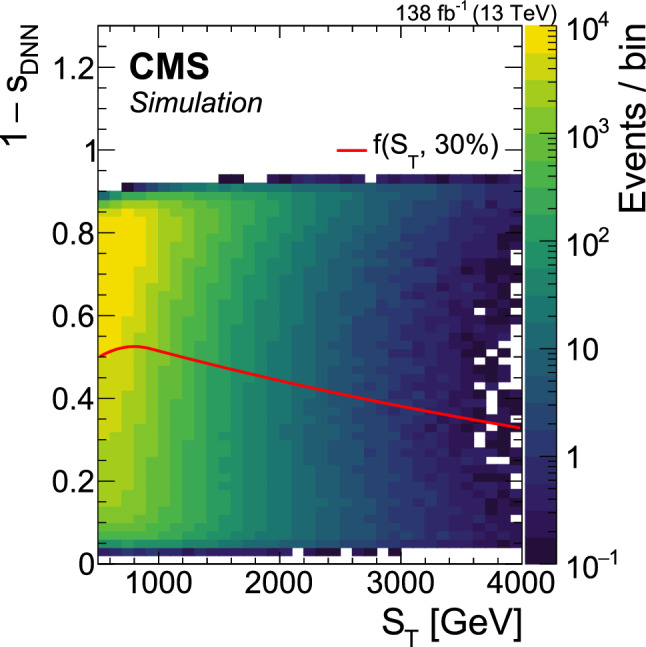
Two-dimensional distribution in $1 - s_{\text {DNN}}$ versus $S_{\text {T}}$ for simulated t t events. The function $f(S_{\text {T}}, 30\%)$ (red line) is determined by specifying a 30% selection efficiency for t t events, i.e., 30% of the t t events are below this function in each bin of $S_{\text {T}}$

Some of the DNN input variables are used to calculate $S_{\text {T}}$, which results in an output strongly correlated with $S_{\text {T}}$. To prevent this and coerce the network to learn auxiliary features that differ between signal and background events, background events are reweighted to have an $S_{\text {T}}$ distribution identical to that of the signal events used in the training. Aside from ensuring balance of the signal and background classes during training, this reduces the correlation between $S_{\text {T}}$ and $s_{\text {DNN}}$. At the same time, it facilitates the exploitation of information other than the $p_{\text {T}}$, such as angular correlations, jet multiplicity, and the substructure of variable-radius jets. The importance of these features for the network decision increases after the $S_{\text {T}}$ reweighting. However, some correlation between $S_{\text {T}}$ and $s_{\text {DNN}}$ remains, which is alleviated in a second decorrelation step. We use the “designing decorrelated taggers” (DDT) technique [72], where we shift $s_{\text {DNN}}$ by an $S_{\text {T}}$-dependent value  for each event,2To determine the function , a set of $s_{\text {DNN}}$ selection thresholds are found in intervals of 100$\,\text {Ge}\hspace{-.08em}\text {V}$ in $S_{\text {T}}$. In each interval, the threshold value corresponds to a given efficiency  for selecting t t background events. To obtain the continuous function , we fit the selection thresholds using an analytic function. The two-dimensional distribution in $S_{\text {T}}$ versus $1-s_{\text {DNN}}$ and the resulting function  are shown in Fig. 3 for .

The resulting score $s_{\text {DDT}}$ is uncorrelated with $S_{\text {T}}$, i.e., a selection based on $s_{\text {DDT}}$ results in a constant t t background efficiency as a function of $S_{\text {T}}$. This is crucial for the background estimation described below. We define a signal region (SR) with $s_{\text {DDT}} >0$ and a validation region (VR) with $s_{\text {DDT}} <0$. While the SR is used in the statistical analysis in the search for a signal, the VR is only used to validate the background estimation presented below. We find that a t t selection efficiency  results in the best sensitivity of this analysis, while ensuring that the split of events between the SR and VR only minimally changes the shape of the $S_{\text {T}}$ distributions for the SM backgrounds. By construction, the SR contains 30% of all t t events after the analysis selection and the VR contains 70%. The efficiency for signal events to enter the SR varies between 55 and 75%, depending on the spin and $m_{\text {t}^{*}}$.

## Background estimation

The SM processes that can result in a final state similar to the one expected from signal events can be divided into two classes: backgrounds containing top quarks, t t and single top quark events, and backgrounds without on-shell top quarks (non-t backgrounds), mostly consisting of events from W +jets, Drell–Yan, QCD multijet, and diboson production.

We estimate backgrounds with top quarks using simulation, for which the correction factors described in Sect. 3 ensure a good description of the data. In addition to these corrections, a reweighting as a function of the parton-level $p_{\text {T}}$ of the top quark is applied to background events. These weights correct for a top quark $p_{\text {T}}$-dependent mismodeling in simulations at NLO precision [73, 74].

**Fig. 4:**
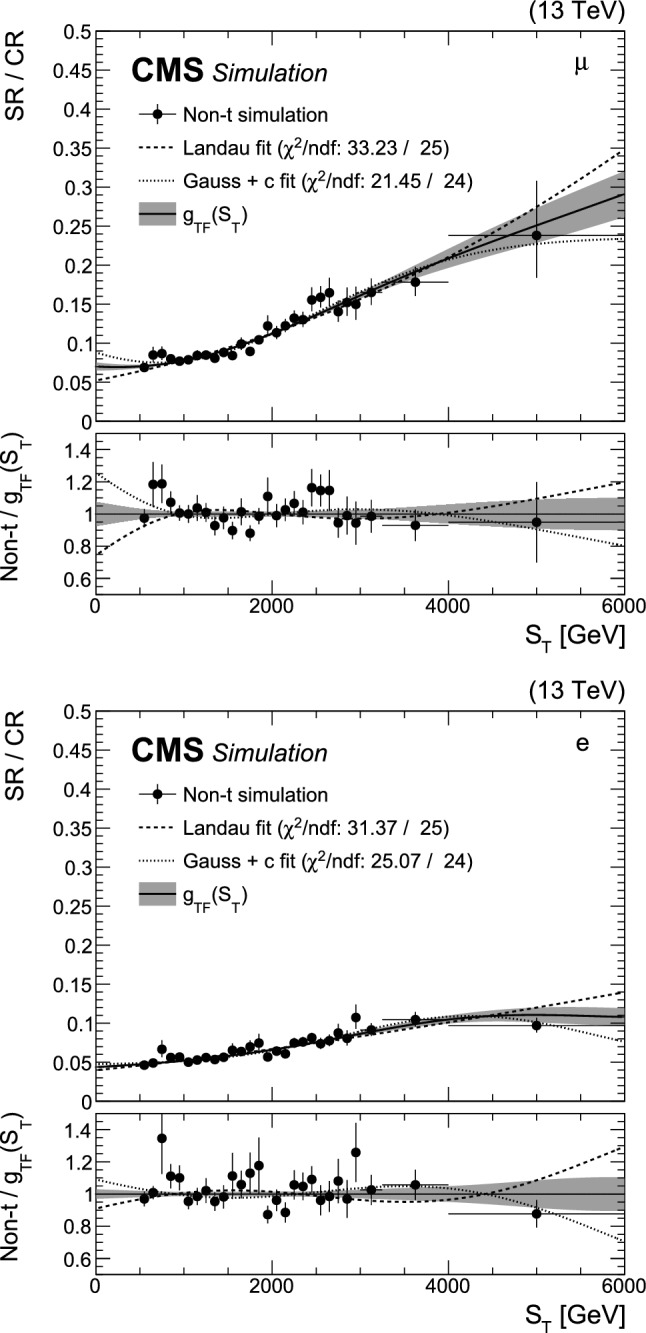
The simulation-based ratios between the $S_{\text {T}}$ distributions in the SRs and CRs for the muon (upper) and electron (lower) channels. Two functions are fit to each ratio, and the final transfer function used for the non-t background estimation is taken to be their average. The statistical uncertainties in the transfer functions are shown as grey bands. In the lower panels, the simulation-based ratios, fit functions, and statistical uncertainties are shown relative to the final transfer function

We estimate the contribution from backgrounds without top quarks with a method based on control samples in data, because the production of weak vector bosons with multiple jets and QCD multijet production are not modeled with sufficient accuracy. We define a control region (CR) that contains events passing all the requirements described in Sect. 4, except for the b jet selection. Only events without a b jet enter the CR. No requirement on $s_{\text {DDT}}$ is imposed in the CR. The CR is enriched in non-t backgrounds, with top quark background contaminations amounting to 10–20%, and is statistically independent of the SR. We use simulated events to construct an $S_{\text {T}}$-dependent ratio between the non-t background contributions in the CR and SR. We fit the ratio with two functions, a Landau distribution and a Gaussian distribution with a constant offset. The average of these two functions then defines a transfer function $g_{\text {TF}} (S_{\text {T}})$. The ratios between the $S_{\text {T}}$ distributions in the SR and CR are shown in Fig. 4, together with the fits of the transfer functions.

**Fig. 5:**
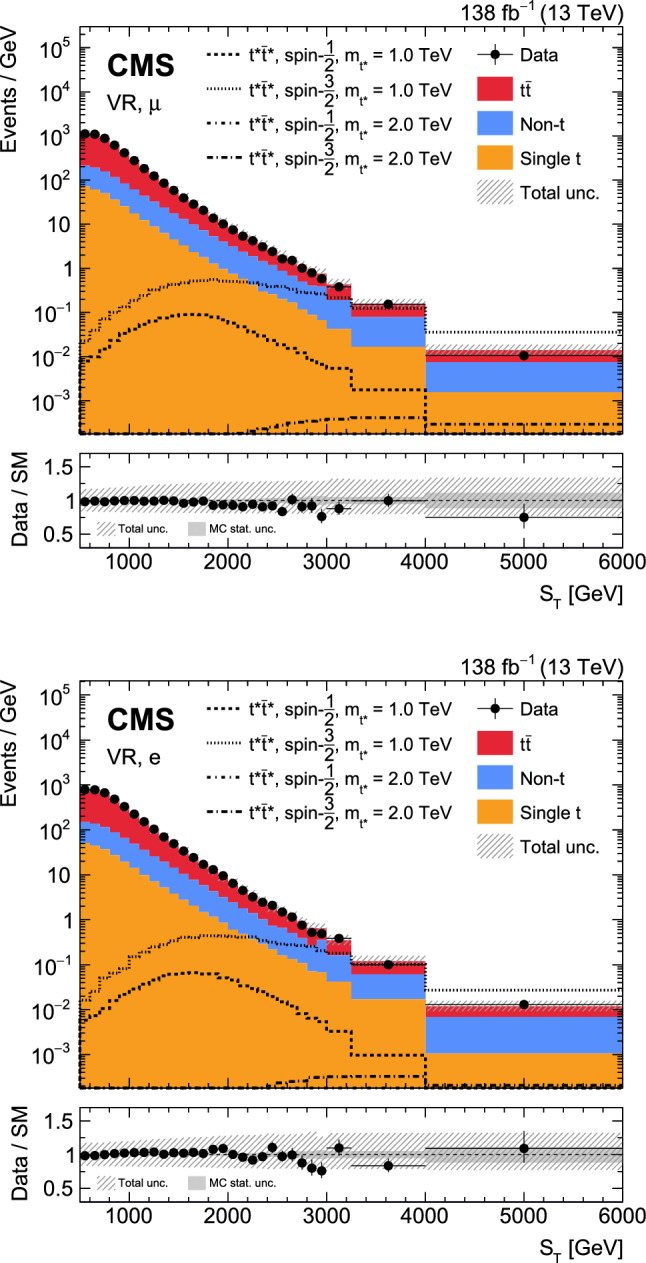
Distributions of $S_{\text {T}}$ in the VR for the muon (upper) and electron (lower) channels. The total uncertainties are shown as hatched bands. The signal distributions are scaled to the cross sections predicted by theory. Ratios of data to the expected backgrounds are shown in the lower panels

The function $g_{\text {TF}} (S_{\text {T}})$ is used to estimate the non-t background contribution in the SR as

3 $$\begin{aligned}  &   N_{\text {SR}}( \text {non}\hbox {-}{\text {t}} \,\text {bkg} )\nonumber \\    &   \quad = g_{\text {TF}} (S_{\text {T}}) \left( N_{ \text {CR} }(\text {data}) - N_{ \text {CR} }(\text {top bkg}) \right) , \end{aligned}$$

where $N_{ \text {CR} }(\text {data})$ and $N_{ \text {CR} }(\text {top bkg})$ are the event counts in the CR for data and for the simulated samples with on-shell top quarks, respectively. The event counts are obtained in intervals of $S_{\text {T}}$. For the muon and electron channels, the number of events in the CR is multiplied by $g_{\text {TF}} (S_{\text {T}})$, after subtracting contributions of simulated top quark backgrounds. Since the composition of non-t backgrounds differs between the muon and electron channels, the method is applied to each channel individually. We verify the estimation of the background in the SR using the VR. Results from the background estimation for the VR are shown in Fig. 5 for the muon and electron channels. The bin widths increase with increasing $S_{\text {T}}$ so that each bin is appropriately populated in data and simulation. The uncertainty arising from the limited size of the simulated background samples becomes non-negligible towards high $S_{\text {T}}$, but its impact on the sensitivity of the analysis is small compared to other uncertainties. The predicted backgrounds describe the measured data within the uncertainties in both channels.

## Systematic uncertainties

Several systematic effects introduce uncertainties affecting the normalization and shape of distributions in $S_{\text {T}}$. We summarize these in the following, split into experimental and theoretical uncertainties. All uncertainties, which can affect either the rate or both the rate and the shape of $S_{\text {T}}$, are included as nuisance parameters in the statistical analysis presented below. Details on the implementation of nuisance parameters are provided in Ref. [75]. To give an estimate of the relevance of the systematic uncertainties, we provide an approximate size of each source for the largest SM contribution it affects.

### Experimental uncertainties

Systematic uncertainties arise from the selection of b jets and the corresponding efficiencies. The b jet selection uncertainties are split into different sources, depending on the jets they affect: jets originating from b quarks, c quarks, or light jets. Furthermore, these are split into statistical and systematic components, where the former are not correlated between eras of data taking, but the latter are. The uncertainty from each source corresponds to variations of up to 10% for t t events. An additional b tagging related uncertainty is connected to normalization differences before and after applying the b tagging correction factors. Separate corrections to the normalization are derived for the electron and muon channels. The differences between these and the correction obtained for a combination of the two channels is taken to be the associated uncertainty. This uncertainty can reach up to 5% for simulated t t events and is correlated between the years of data taking.

The impact of uncertainties associated with the jet energy scale and jet energy resolution corrections on small radius jets, variable-radius jets and ${\textbf{p}}_{\text {T}}^{\hspace{1.66656pt}\text {miss}}$ is estimated by varying the corresponding corrections within their uncertainties. The resulting uncertainty is correlated between all affected objects and treated as uncorrelated between years. This uncertainty has an impact of up to 4% in t t events.

Uncertainties connected to correction factors for the lepton reconstruction, identification, isolation, and triggers are of the order of 1% or smaller for t t events, and are not correlated between years of data taking. An uncertainty in the correction factor accounting for the 2016 and 2017 trigger inefficiency is considered as well.

The total inelastic cross section of 69.2$\,\text {mb}$, used to correct the pileup distribution in simulation, is varied by ${\pm }4.6\%$ [48], resulting in an uncertainty of around 2% for t t events.

A total uncertainty of 1.6% in the integrated luminosity of 138$\,\text {fb}^{-1}$ is considered [76–78]. This uncertainty affects only the normalization of simulated samples.

Another source of uncertainty, specific to this analysis, arises from the DNN decorrelation procedure. We take the residual differences between the shapes of the $S_{\text {T}}$ distributions in the SR and VR for top quark backgrounds as an estimate of the associated uncertainty. The resultant impact corresponds to variations of up to 10% for t t events.

Separate uncertainties are considered on the results of the data-driven background estimation. The procedure is repeated for each of the two functions used to construct $g_{\text {TF}} (S_{\text {T}})$. The resulting distributions are used as a systematic variation around the nominal non-t background prediction. The effect is treated as fully correlated between years and is below 8% for the non-t background. The effect of the statistical uncertainty in $g_{\text {TF}} (S_{\text {T}})$ amounts to around 5%. We estimate the systematic uncertainty connected to the b tagging veto in the definition of the CR by repeating the background estimation for variations of the b tagging corrections, where the individual sources are combined. The resulting uncertainty can reach up to 15% for the non-t background and is treated as fully correlated between years.

In summary, the dominant experimental systematic uncertainties in this analysis are from the b tagging corrections and the decorrelation procedure for the background estimation.

### Theoretical uncertainties

The largest systematic uncertainty in the modeling of SM background processes results from missing higher-order effects in the simulation. We estimate this effect by varying the renormalization and factorization scales in the simulation by factors of 2 and 1/2. An envelope is constructed from the possible variations, except for variations where one scale is varied by 2 and the other by 1/2. This uncertainty is taken to be fully correlated between data-taking years. It includes a normalization effect covering uncertainties in the production cross sections of the SM background processes and varies between 20 and 60% for t t events, with the larger values at high $S_{\text {T}}$. For signal samples, we remove uncertainties in the normalization from scale variations and consider only acceptance and shape effects.

The uncertainty in the correction of the $p_{\text {T}}$ of the parton-level top quark is estimated from the difference in the $S_{\text {T}}$ distribution before and after applying this correction. For t t events, it amounts to below 20%.

An uncertainty resulting from the choice of PDFs is obtained using the standard deviation of 100 different MC replicas, following the procedure described in Ref. [79]. The uncertainty has an unimportant effect on the analysis. For signal samples, only the acceptance and shape effects are considered for the PDF uncertainties.

Other sources of uncertainties related to the modelling of background processes, like uncertainties in the simulation of initial and final state radiation, the matching of matrix elements to the parton shower, or the modelling of hadronization, are small compared to the ones considered here.

## Results

The $S_{\text {T}}$ distribution in the SR, measured in the muon and electron channels, is the main observable of this analysis. The backgrounds from t t and single top quark production are taken from simulation, with the appropriate correction factors applied. The $S_{\text {T}}$ distributions of the non-t backgrounds are extrapolated from the CR. Signal distributions are taken from simulations. We combine all years of data and include the uncertainties described in Sect. 7 as nuisance parameters.

A statistical analysis is performed to probe for the existence of $\text {t}^{*} \bar{{\text {t}}}^{*}$ production with a binned maximum likelihood fit. The modified frequentist approach [80–82], known as the $\text {CL}_\text {s}$ criterion with the profile likelihood ratio as the test statistic, is used in this search to set 95% confidence level ($\text {CL}$) upper limits on the product of the production cross section for ${\text {p}} {\text {p}} \rightarrow \text {t}^{*} \bar{{\text {t}}}^{*}$ and the branching fraction squared $\mathcal {B} ^2(\text {t}^{*} \rightarrow {\text {t}} {\text {g}} )$. We use the asymptotic approximation to the profile likelihood test statistic [83]. The statistical analysis is performed using the CMS statistical analysis tool Combine [75], which is based on the RooFit [84] and RooStats [85] frameworks.

**Fig. 6:**
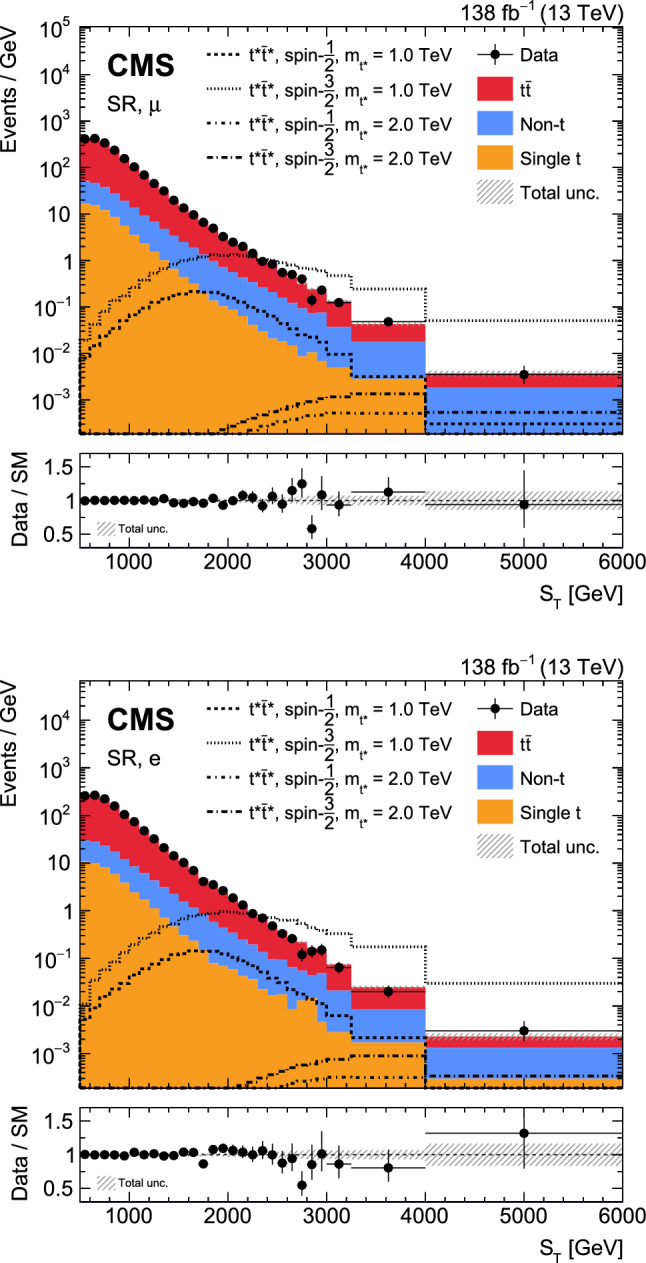
Distributions in $S_{\text {T}}$ in the SR for the muon (upper) and electron (lower) channels, after a background-only fit to the data. The signal distributions are scaled to the cross section predicted by the theory. The hatched bands show the post-fit uncertainty band, combining all sources of uncertainty. The ratio of data to the background predictions is shown in the panels below the distributions

**Fig. 7:**
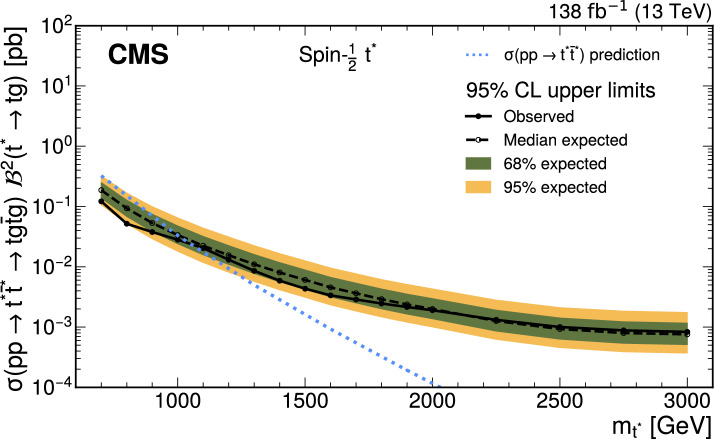
Expected and observed 95% $\text {CL}$ upper limits on the product of the $\text {t}^{*} \bar{{\text {t}}}^{*}$ production cross section and the branching fraction squared $\mathcal {B} ^2(\text {t}^{*} \rightarrow {\text {t}} {\text {g}} )$ for a spin-1/2 $\text {t}^{*}$as a function of $m_{\text {t}^{*}}$. The inner (green) and outer (yellow) bands give the central probability intervals containing 68 and 95% of the expected upper limits under the background-only hypothesis. The cross section predicted by theory, following the EFT approach introduced in Ref. [26], is shown as a dotted line, assuming $\mathcal {B} (\text {t}^{*} \rightarrow {\text {t}} {\text {g}} )=1$

**Fig. 8:**
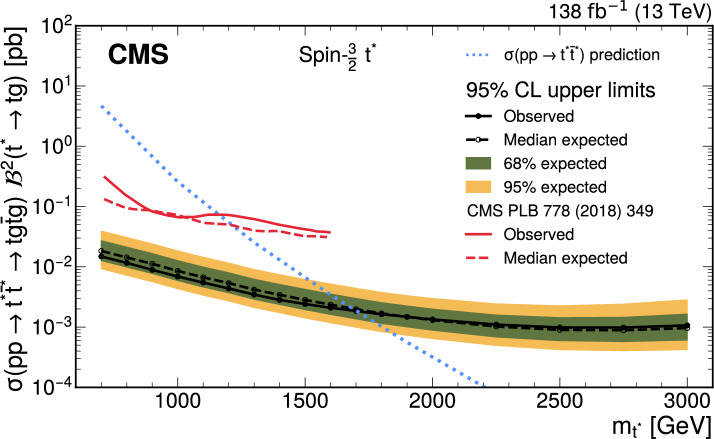
Expected and observed 95% $\text {CL}$ upper limits on the product of the $\text {t}^{*} \bar{{\text {t}}}^{*}$ production cross section and the branching fraction squared $\mathcal {B} ^2(\text {t}^{*} \rightarrow {\text {t}} {\text {g}} )$ for a spin-3/2 $\text {t}^{*}$as a function of $m_{\text {t}^{*}}$. The inner (green) and outer (yellow) bands give the central probability intervals containing 68 and 95% of the expected upper limits under the background-only hypothesis. The cross section predicted by theory, following the EFT approach introduced in Ref. [26], is shown as a dotted line, assuming $\mathcal {B} (\text {t}^{*} \rightarrow {\text {t}} {\text {g}} )=1$. The results of the previous CMS analysis [30], using data corresponding to an integrated luminosity of 35.9$\,\text {fb}^{-1}$, are shown as well

We show the $S_{\text {T}}$ distributions in the SR for the muon and electron channels in Fig. 6 after the background-only maximum likelihood fit to the data. No significant deviation from the SM predictions is observed. The resulting upper limits on the product of the production cross section and the branching fraction squared are presented in Figs. 7 and 8 for spin-1/2 and spin-3/2 resonances, respectively. For the spin-1/2 case, the limits are found to be between 120$\,\text {fb}$ (190$\,\text {fb}$ expected) for $m_{\text {t}^{*}} =700\,\text {Ge}\hspace{-.08em}\text {V}$, and 0.8$\,\text {fb}$ (0.8$\,\text {fb}$ expected) for $m_{\text {t}^{*}} =3000\,\text {Ge}\hspace{-.08em}\text {V}$. For the spin-3/2 case, the upper limits are between 15$\,\text {fb}$ (18$\,\text {fb}$ expected) at 700$\,\text {Ge}\hspace{-.08em}\text {V}$ and 1.0$\,\text {fb}$ (0.9$\,\text {fb}$ expected) at 2750$\,\text {Ge}\hspace{-.08em}\text {V}$.

As expected from the differences in the $S_{\text {T}}$ distributions between spin-1/2 and spin-3/2 $\text {t}^{*}$signals, the analysis is more sensitive to spin-3/2 signals at low $m_{\text {t}^{*}}$. At high $m_{\text {t}^{*}}$, the sensitivity is comparable, with the analysis being slightly more sensitive to spin-1/2 signals. This is a result of the efficiency of the DNN-based SR definition, which decreases with increasing $m_{\text {t}^{*}}$. This effect is more pronounced for spin-3/2 than for spin-1/2 $\text {t}^{*}$signals.

For both spin scenarios, we compare the results to theory predictions of the $\text {t}^{*} \bar{{\text {t}}}^{*}$ pair production cross section. These are determined as described in Sect. 2, using an EFT approach to describe the interaction of the $\text {t}^{*}$and SM particles. We assume a branching fraction of 100% for the $\text {t}^{*} \rightarrow {\text {t}} {\text {g}}$ decay.

The existence of a spin-1/2 $\text {t}^{*}$is excluded up to $m_{\text {t}^{*}}$ values of 1050$\,\text {Ge}\hspace{-.08em}\text {V}$, with 990$\,\text {Ge}\hspace{-.08em}\text {V}$ expected. These are the first mass exclusion limits on a spin-1/2 $\text {t}^{*}$in the $\text {t}^{*} \rightarrow {\text {t}} {\text {g}}$ decay channel at 13 $\,\text {Te}\hspace{-.08em}\text {V}$, made possible by the improvements in this analysis compared to the previous analysis [30]. For the spin-3/2 case, the lower mass limit is considerably higher because of the higher predicted cross section and the better sensitivity of the analysis to spin-3/2 $\text {t}^{*}$resonances below 2$\,\text {Te}\hspace{-.08em}\text {V}$. The existence of a spin-3/2 $\text {t}^{*}$is excluded below $m_{\text {t}^{*}}$ of 1700$\,\text {Ge}\hspace{-.08em}\text {V}$, with 1690$\,\text {Ge}\hspace{-.08em}\text {V}$ expected.

When comparing the exclusion limits to the ones from the previous CMS result, based on 13$\,\text {Te}\hspace{-.08em}\text {V}$ data corresponding to an integrated luminosity of 35.9$\,\text {fb}^{-1}$, a substantial improvement is visible. The increased size of the analyzed data set yields an improvement in sensitivity to the cross section limit of about a factor of two. In addition, the sensitivity is increased by improvements of the analysis strategy. We impose looser lepton and jet multiplicity requirements and use variable-radius jets to access the Lorentz-boosted $\text {t}^{*}$decay products. This, combined with the addition of an event classification DNN and the usage of $S_{\text {T}}$ instead of a reconstruction of the $\text {t}^{*}$system, leads to an increased signal efficiency: the fraction of signal events reaching the SR is about five times higher compared to the previous analysis. In contrast, background yields in the SR only increase by about 10%. Overall, we achieve an increase in sensitivity by about a factor of five on top of the factor of two from the larger data set, resulting in a total improvement in sensitivity by about an order of magnitude compared to the previous analysis.

## Summary

A search for the pair production of heavy top-quark partners $\text {t}^{*}$has been presented, where the $\text {t}^{*}$couples predominantly to gluons and decays to a top quark and a gluon, $\text {t}^{*} \rightarrow {\text {t}} {\text {g}}$. Both spin-1/2 and spin-3/2 resonances are considered. The analysis uses 13$\,\text {Te}\hspace{-.08em}\text {V}$ proton–proton collision data collected by the CMS experiment between 2016 and 2018, corresponding to an integrated luminosity of 138$\,\text {fb}^{-1}$. The final state analyzed consists of a lepton with high transverse momentum, missing transverse momentum and several jets. A deep neural network is used to identify potential signal events. With a two-step decorrelation procedure, independence of the deep neural network output from the main observable $S_{\text {T}}$ has been achieved, where $S_{\text {T}}$ is the scalar sum of the transverse momenta of the selected lepton and jets, and the missing transverse momentum. No statistically significant deviation from the background prediction was found. Upper limits at 95% confidence level are derived on the product of the $\text {t}^{*} \bar{{\text {t}}}^{*}$ production cross section and branching fraction squared for $\text {t}^{*} \rightarrow {\text {t}} {\text {g}}$. These are between 120 and 0.8$\,\text {fb}$ for a spin-1/2 $\text {t}^{*}$and between 15 and 1.0$\,\text {fb}$ for a spin-3/2 $\text {t}^{*}$, depending on the $\text {t}^{*}$mass. A comparison of these limits with the theory predictions results in mass limits for the $\text {t}^{*}$resonances, where the existence of a spin-1/2 $\text {t}^{*}$is excluded below a mass of 1050$\,\text {Ge}\hspace{-.08em}\text {V}$ and for a spin-3/2 $\text {t}^{*}$below a mass of 1700$\,\text {Ge}\hspace{-.08em}\text {V}$. These are the most stringent limits to date and the first exclusion limit for a spin-1/2 $\text {t}^{*}$resonance at 13 $\,\text {Te}\hspace{-.08em}\text {V}$. The results also substantially improve the spin-3/2 exclusion limits compared to previous results.

## Data Availability

Release and preservation of data used by the CMS Collaboration as the basis for publications is guided by the CMS data preservation, re-use and open access policy.

## Code Availability

The CMS core software is publicly available on GitHub (https://github.com/cms-sw/cmssw).
